# AVATAR therapy for medication-resistant auditory hallucination in patients with psychosis: a systematic review and meta-analysis

**DOI:** 10.1038/s41537-025-00671-5

**Published:** 2025-12-13

**Authors:** Tien-Wei Hsu, Ping-Tao Tseng, Chih-Wei Hsu, Fu-Chi Yang, Te-Chang Changchien, Yu-Hsuan Lin, Chih-Sung Liang

**Affiliations:** 1 Department of Psychiatry, E-DA Dachang Hospital, I-Shou University, Kaohsiung, Taiwan; 2 Department of Psychiatry, E-DA Hospital, I-Shou University, Kaohsiung, Taiwan; 3 Graduate Institute of Clinical Medicine, College of Medicine, Kaohsiung Medical University, Kaohsiung, Taiwan; 4 School of Medicine, College of Medicine, I-Shou University, Kaohsiung, Taiwan; 5 Institute of Biomedical Sciences, National Sun Yat-sen University, Kaohsiung, Taiwan; 6 Department of Psychology, College of Medical and Health Science, Asia University, Taichung, Taiwan; 7 Prospect Clinic for Otorhinolaryngology & Neurology, Kaohsiung, Taiwan; 8 Institute of Precision Medicine, National Sun Yat-sen University, Kaohsiung City, Taiwan; 9 Department of Psychiatry, Kaohsiung Chang Gung Memorial Hospital and Chang Gung University College of Medicine, Kaohsiung, Taiwan; 10 Department of Neurology, Tri-Service General Hospital, National Defense Medical Centre, Taipei, Taiwan; 11 Institute of Population Health Sciences, National Health Research Institutes, Miaoli County, Taiwan; 12 Department of Psychiatry, College of Medicine, National Taiwan University, Taipei, Taiwan; 13 Department of Biomedical Sciences and Engineering, National Central University, Taoyuan City, Taiwan; 14 Department of Psychiatry, Tri-Service General Hospital, National Defense Medical Centre, Taipei, Taiwan; 15 Department of Psychiatry, Beitou Branch, Tri-Service General Hospital, Taipei, Taiwan

**Keywords:** Schizophrenia, Psychosis

## Abstract

Auditory hallucination (AH) is a distressing and disabling symptom in patients with schizophrenia spectrum disorder, particularly in those who do not respond to antipsychotics. The aim of this study is to examine the efficacy of AVATAR (Audio Visual Assisted Therapy Aid for Refractory auditory hallucinations) therapy for medication-resistant AH in patients with schizophrenia spectrum disorder. A systematic search was conducted across five major databases for randomized controlled trials (RCTs) investigating AVATAR therapy for patients with medication-resistant AH, with control conditions such as treatment-as-usual (TAU), cognitive behavioral therapy (CBT), or supportive therapy. The primary outcome was AH severity improvement, measured by the Psychotic Symptom Rating Scale-Auditory Hallucination. The secondary outcomes were positive and negative symptoms (assessed using the Positive And Negative Syndrome Scale), quality of life, depression, anxiety, and acceptance (all-cause discontinuation). Additionally, we evaluated the long-term efficacy by examining the sustained effects after treatment discontinuation. Six RCTs (*n* = 675; 64.7% male; mean age 39.4 [SD 4.8] years) were included. AVATAR therapy was associated with AH improvement (mean difference [MD], −2.97; 95%CI: −4.03, −1.90) and positive symptoms reduction (MD, −1.13; 95%CI: −2.14, −0.11) compared to controls. It also showed efficacy in improving depressive symptoms, anxiety, and quality of life, with small-to-medium effect sizes. The three-month follow-up effects remained consistent with treatment effect at study endpoints across all outcomes. The all-cause discontinuation rate did not differ between AVATAR therapy and controls. Given its potential benefits, clinicians may consider implementing AVATAR therapy for patients with medication-resistant symptoms. However, the development of standardized treatment protocols or manuals is essential to ensure treatment fidelity and guide future clinical and research applications.

## Introduction

Treatment-resistant schizophrenia (TRS) affects approximately 20-30% of individuals with schizophrenia and is characterized by persistent psychotic symptoms despite adequate trials of antipsychotic medications^1^. Persistent auditory hallucinations (AH), a common feature of TRS, could severely disrupt daily life and well-being by causing emotional distress, triggering anxiety and depressive symptoms, increasing suicide risk, and impairing work, relationships, and daily functioning^2–6^. A U.S. psychiatrist survey found that persistent hallucinatory behavior was the top symptom prompting therapeutic changes in TRS (~63.9%), reflecting its strong clinical impact and burden^7^. the etiology of auditory hallucinations in schizophrenia is multifactorial, involving genetic predispositions, neurotransmitter imbalances (e.g., glutamatergic dysfunction), and structural and functional abnormalities in auditory and language-related brain networks^8^. Exploring and implementing non-pharmacological therapies as complementary treatment is essential for improving outcomes in patients with TRS. Among these, cognitive behavioral therapy (CBT) is a widely studied intervention for schizophrenia with AH. CBT helps patients challenge and reframe distressing thoughts, reducing the emotional and behavioral impact of AH^9–11^. However, its effectiveness may be limited in patients with schizophrenia, particularly those with negative symptoms or cognitive impairments, because they may struggle with the motivation or cognitive engagement required for active participation^12,13^.

Virtual reality (VR), incorporating both avatars (user-controlled figures) and agents (computer-controlled figures), is increasingly used in psychiatric treatment, offering immersive environments that enhance therapeutic engagement. These immersive technologies, especially when involving interactive virtual agents or avatars, provide controlled, safe, and tailored interventions—especially for conditions such as social anxiety disorder, agoraphobia, post-traumatic stress disorder, and substance use disorders^14^. In 2006, Ku et al. conducted a pilot study to investigate whether virtual avatars could be perceived as human by patients with schizophrenia and used to assess their behavioral characteristics in brief interactions^15^. The study found that interpersonal distance in the virtual environment was negatively correlated with the negative symptoms, particularly blunted affect and poor rapport, suggesting that the avatars effectively elicited patients’ behavioral traits^15^. In 2013, Leff et al. introduced AVATAR therapy (Audio Visual Assisted Therapy Aid for Refractory auditory hallucinations), a computer-based interface adaptation of CBT for medication-resistant AH. Patients collaborate with therapists to create a digital avatar that represents the distressing voice, enabling them to engage in structured, therapist-guided dialogues aimed at reducing the power and distress associated with auditory hallucinations. The therapy integrates CBT techniques, such as cognitive restructuring, voice-challenging strategies, and emotion regulation, within these interactive sessions^16^. Because patients with cognitive impairments or negative symptoms often struggle to engage in traditional CBT, AVATAR therapy offers a more immersive and engaging alternative that may improve participation and adherence. In 2020, Cochrane Reviews published a systematic review and meta-analysis on the use of AVATAR therapy for schizophrenia and related disorders. However, the review included only three studies (two of which were unpublished then) and did not assess long-term efficacy^17^. To date, several clinical trials—including newly published ones— have investigated AVATAR therapy for medication-resistant AH, and a recent scoping review reported promising results^18^.

To address this gap, this systemic review and meta-analysis examined the efficacy of AVATAR therapy for patients with medication-resistant AH. Additionally, we assessed its long-term efficacy after discontinuation to determine the durability of treatment benefits. By synthesizing existing evidence, we believe this study aims to clarify AVATAR therapy’s clinical utility and inform its integration into schizophrenia treatment strategies.

## Methods

Prior to data analysis, we registered our protocol on PROSPERO (CRD42025639959). The study adhered to the Preferred Reporting Items for Systematic Reviews and Meta­analyses (PRISMA)^19^, which can be found in Appendix 1.

### Eligibility criteria

**Fig. 1:**
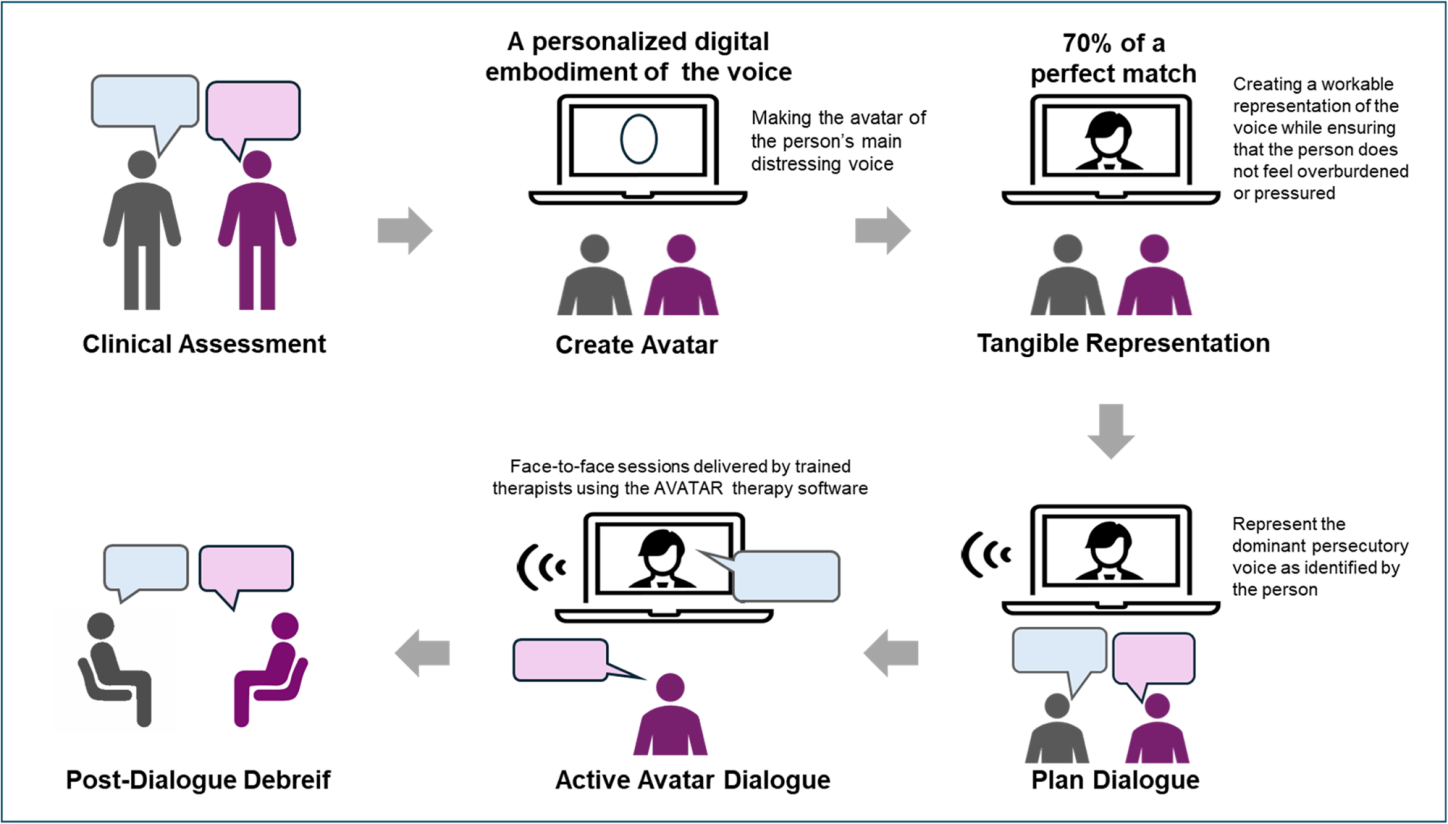
Illustration of Avatar therapy.

We included randomized controlled trials (RCTs) of adult patients with schizophrenia spectrum disorder who had persistent medication-resistant auditory hallucinations. The included studies were required to compare AVATAR therapy with control interventions. AVATAR therapy represents a novel therapeutic approach in which individuals with schizophrenia experiencing persecutory auditory hallucinations engage in face-to-face dialogue with a digital representation that matches their persecutory voice in pitch and tone. In this therapeutic process, a therapist alternates between speaking as the avatar and as a therapist, facilitating a dialogue where patients gradually gain increased power and control over their voice experiences (Fig. 1). This innovative integration of digital technology with psychological intervention provides a unique way to directly engage with and modify the voice-hearing experience. The control group could receive either passive (e.g., treatment as usual or waitlist) or active (e.g., cognitive behavioral therapy or other virtual reality intervention) treatments. These criteria were selected to examine the isolated efficacy of AVATAR therapy compared to non-AVATAR interventions and to explore whether AVATAR therapy provided additional benefits.

The exclusion criteria were: (i) non-randomized studies, (ii) studies without specific measurements for auditory hallucination, (iii) studies with patients who had not received adequate antipsychotic treatment, and (iv) studies not published in peer-reviewed journals.

### Data sources and search

Two reviewers independently searched MEDLINE, the Cochrane Central Register of Controlled Trials (CENTRAL), EMBASE, and PubMed without language restrictions, using the search terms (voice OR auditory hallucination) AND (virtual real* OR AVATAR OR virtual environ* OR virtual character*). All literature deposited in any given database from its inception to 17 February 2025, was searched. We also reviewed the reference lists of the included studies and related systematic reviews.

### Study selection

Two reviewers independently screened titles, abstracts, and full-text articles. Disagreements were resolved through discussion and, if necessary, by consulting the corresponding authors. Appendix 2 demonstrates the complete search strategies and appendix 3 shows the reasons for exclusion.

### Data Extraction and Outcome Definition

Two reviewers independently extracted the data and discrepancies were resolved by consensus and, when necessary, by consulting the corresponding authors. WebPlot Digitizer (https://apps.automeris.io/wpd/) was used to extract numerical data from the figures. The following data were extracted from each study: authorship, year of publication, country of origin, and study design. For participant characteristics, we collected information on diagnosis type, sample size, mean age with standard deviation (SD), and sex distribution (percentage of females). Intervention details included the number and duration of AVATAR therapy sessions. For comparison groups, we documented the type of control interventions, such as treatment as usual, cognitive behavioral therapy, or waitlist control.

The primary outcome was the score of Psychotic Symptom Rating Scale-Auditory Hallucination. The secondary outcomes were the Beliefs About Voices Questionnaire, the Positive And Negative Syndrome Scale, quality of life, depression, anxiety, and all-cause discontinuation. If the study provided the follow-up data after AVATAR discontinuation, we also extracted the follow-up data.

### Quality assessment

The risk of bias (RoB) was assessed using the Cochrane risk-of-bias tool 2 (RoB-2) for randomized trials. This tool evaluates five domains of potential bias: bias arising from the randomization process, bias due to deviations from intended interventions, bias due to missing outcome data, bias in outcome measurement, and bias in the selection of reported results. Two authors independently conducted this assessment, and discrepancies were resolved by consulting the corresponding authors.

### Data synthesis

We applied a random-effects mode with the restricted maximum likelihood method to account for heterogeneity across the included studies. The mean difference (MD) was and 95% confidence intervals (CI) were calculated. We summarized outcomes reported as either change from baseline or post-treatment separately, following standard practice. If both were reported, we used change from baseline. Change from baseline represents the difference in a variable before and after AVATAR treatment, using the pre-intervention level as a reference. In contrast, post-treatment measures are observations made after the intervention. Additionally, to facilitate comparisons with previous studies reporting standardized mean differences (SMD), we also calculated SMD. The analysis was conducted using RStudio, version 4.3.0 (R Foundation for Statistical Computing). Specifically, we used the package meta to calculate the meta-analysis and create the forest plots for all outcomes with sufficient and adequate outcome data. The sample size for each study arm was extracted based on the initial sample size at randomization (intention-to-treat analysis) to maintain the balanced consideration of potential confounders inherent in RCTs. Heterogeneity was evaluated using the Q statistic and I^2^ statistics. Publication bias was assessed with funnel plots and the Egger test if more than ten studies were included. Leave-one-out analyses were conducted as sensitivity tests.

## Results

### Study characteristics

Our searches resulted in 52 potentially relevant citations (eFigure 1). The complete search strategies and reasons for the exclusion of certain studies can be found in appendix 2 and 3. After removing duplicates, we included 6 RCTs^16,20–24^. eTable 1 shows the characteristics of included studies. These studies included 675 participants with mean age of 39.37 years (4.77), and 64.74% were male. Three studies^16,20,22^ were assessor-blinded design, and the other three^21,23,24^ were open-label. Three studies^21,23,24^ used a VR interface, while the other three^16,20,22^ used a computer-based interface.

### Quality of evidence

Overall risk of bias was Moderate in 4 studies^16,20,22,23^ and High in 2 studies^21,24^. Ratings for individual domains for each study are provide in eFigure 2 and 3. The proportions of studies with high, some concerns, and low ROB for the individual items of AVATAR trials were as follows: 0/6, 2/6, and 4/6 for randomization; 2/6, 4/6, and 0/6 for deviations from intended interventions; 0/6, 3/6, and 3/6 for missing outcome data; 0/6, 2/6, and 4/6 for measurements of outcomes; 0/6, 5/6, and 1/6 for selection of reported results.

### Primary outcome- auditory hallucination severity

**Fig. 2:**
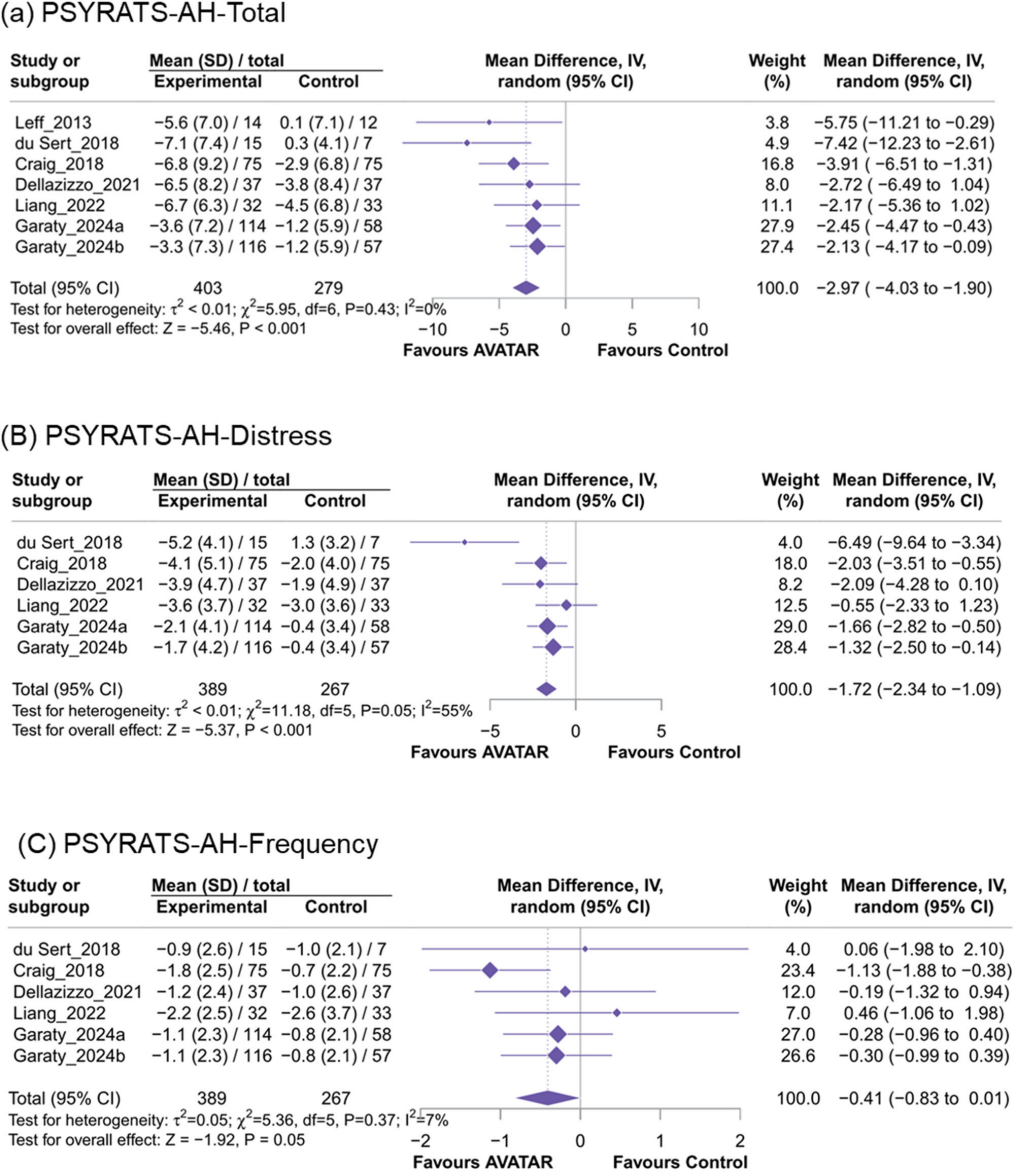
Forest plot of meta-analysis of mean changes of the primary outcome of the Psychotic Symptoms Rating Scale-Auditory Hallucinations Scale. **A** PSYRATS-AH total scale (**B**) Distress subscale of PSYRATS-AH (**C**) Frequency subscale of PSYRATS-AH.

AVATAR therapy was associated with significant improvement in AH severity compared to the controls, with a mean PSYRATS-AH-total scale difference of −2.97 points (Fig. 2A, the number of included studies [*k*]=6, 95% CI = −4.03 to −1.90) and a SMD of −0.40 (95% CI = −0.56 to −0.25). No significant heterogeneity was observed (*I*^2^ = 0%). For the distress (Fig. 2B) and frequency (Fig. 2C) subscales, AVATAR therapy remained more effective than controls in reducing distress (*k* = 6, MD = −1.72, 95% CI = −2.34 to −1.09, *I*^2^ = 55%; SMD = −0.40, 95% CI = −0.56 to −0.24), while the 95% CI of effect on the frequency crossed zero (*k* = 6, MD = −0.41, 95% CI =−0.83 to 0.01, *I*^2^ = 7%; SMD = −0.16, 95% CI = −0.35 to 0.02).

### Secondary outcomes

**Fig. 3:**
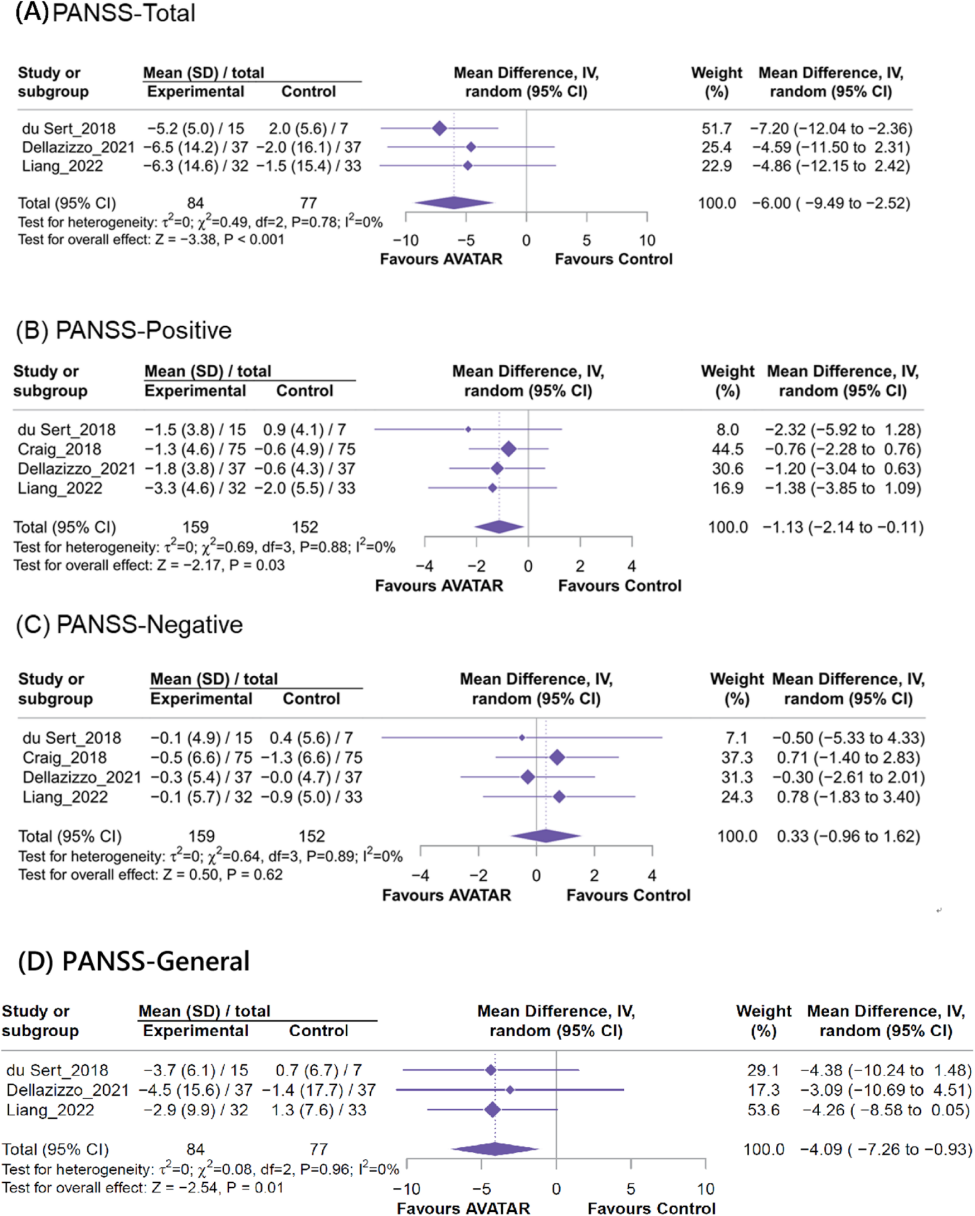
Forest plot of meta-analysis of mean changes of the overall psychotic symptoms assessed by PANSS. **A** PANSS total score (**B**) PANSS positive score (**C**) PANSS negative score (**D**) PANSS general score.

For overall psychotic symptoms, AVATAR therapy was associated with significant improvement in overall psychotic symptoms measured by PANSS total score (Fig. 3A, *k* = 3, MD = −6.00, 95% CI = −9.49 to −2.52, *I*^2^ = 0%; SMD = −0.41, 95% CI = −0.73 to −0.10) than controls. Additionally, AVATAR therapy was also associated with reduction in positive symptoms measured by PANSS positive score (Fig. 3B, *k* = 4, MD = −1.13, 95% CI = −2.14 to −0.11, *I*^2^ = 0%; SMD = −0.24, 95%CI = −0.46 to −0.01) than controls. However, we did not find significant difference between AVATAR and control therapies in negative symptoms measured by PANSS negative score (Fig. 3C, *k* = 4, MD = 0.33, 95%CI = −0.96 to 1.62, *I*^2^ = 0%; SMD = 0.06, 95% CI = −0.16 to 0.29). For PANSS general psychopathology subscale, AVATAR therapy showed significant improvement compared to the control therapies (Fig. 3D, *k* = 3, MD = −4.09, 95% CI = −7.26 to –0.93, *I*^2^ = 0%; SMD = −0.36, 95%CI = −0.67 to −0.04).

**Fig. 4:**
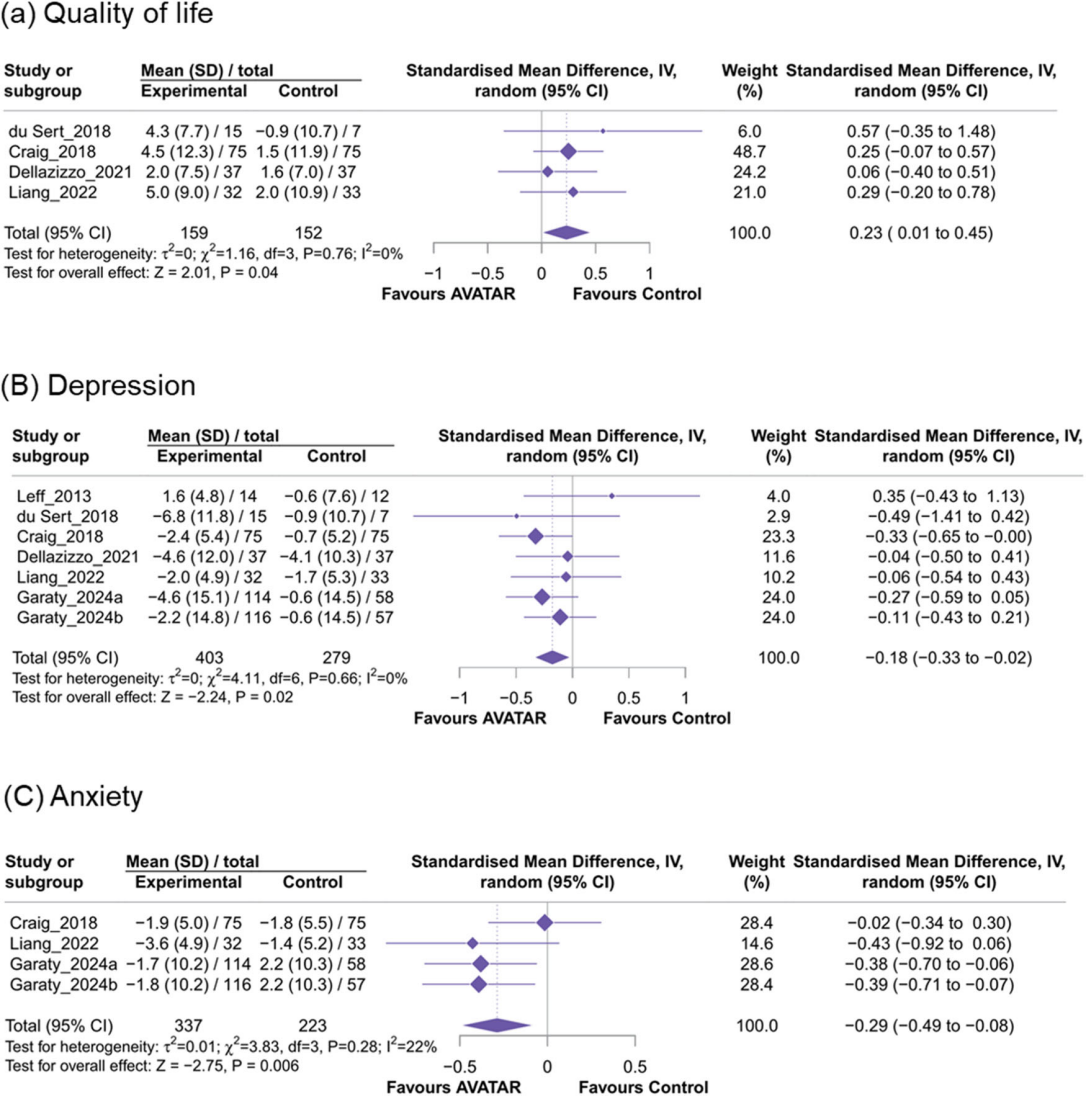
Forest plot of meta-analysis of standardized mean difference in other secondary outcomes. **A** Quality of life (**B**) Depression (**C**) Anxiety.

AVATAR therapy was also associated with improved quality of life (k = 4, standard mean difference [SMD] = 0.23, 95%CI = 0.01 to 0.45, I^2^ = 0%, Fig. 4a) and reduction in depressive symptoms (k = 7, SMD = -0.18, 95%CI = -0.33 to -0.02, I^2^ = 0%, Fig. 4B) and anxiety symptoms (k = 4, SMD = -0.29, 95%CI = -0.49 to -0.08, I^2^ = 22%, Fig. 4C) compared to controls in patients with medication-resistant AH.

There was no significant difference between AVATAR therapy and control therapies in patients with medication-resistant AH regarding beliefs about voices, including total score, malevolence, omnipotence, benevolence, and persecutory belief subscales (malevolence plus omnipotence) (eFigure 4 to 8). Similarly, AVATAR therapy showed no significant difference in treatment acceptance compared to control therapies (*k* = 6, risk ratio = 1.14, 95% CI = 0.78 to 1.67, *I*^2^ = 1%, eFigure 9).

### Post treatment 3-month follow-up effect

**Fig. 5:**
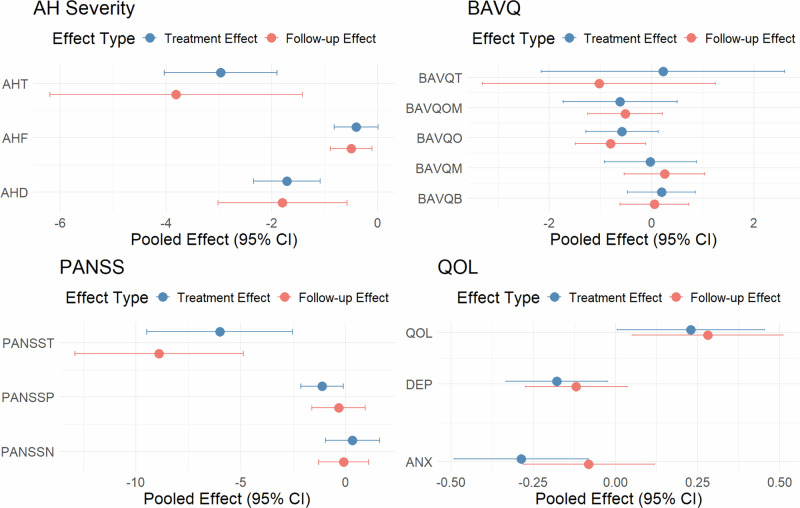
Treatment effects and follow-up effects for all the primary and secondary outcomes. AH, auditory hallucination (T, total scale; F, frequency subscale; D, distress subscale); BAVQ, Beliefs About Voices Questionnaire (T, total scale; OM, Omnipotence + Malevolence; O, Omnipotence; M, Malevolence; B, Benevolence); PANSS, Positive And Negative Syndrome Scale (T, total score; P, positive score; N, negative score) QOL, quality of life; DEP, depressive symptoms; ANX, anxiety symptoms; CI, confidence interval.

For AH severity, the post-treatment follow-up effect of AVATAR therapy was even slightly better than the treatment effect at study endpoints (Fig. 5). For secondary outcomes, the post-treatment follow-up effects were generally comparable to treatment effects at study endpoints (Fig. 5). Notably, AVATAR therapy showed sustained improvement in the overall psychotic symptoms measure by the PANSS total scale, the beliefs about voices-omnipotence subscale, and quality of life compared to control therapies. However, AVATAR therapy was not associated with significant follow-up effect in other beliefs about voices subscales except for the omnipotence subscale, treatment follow-up efficacy in measurements in beliefs about voices except omnipotence subscale, the PANSS positive and negative scales, depressive symptoms, or anxiety symptoms. Detailed results of the meta-analyses of follow-up effect were presented in eFigure 10 to 23.

### Subgroup analyses: VR vs. computer screen

Subgroup analysis revealed that both computer-screen-based (SMD = –0.40, 95% CI: –0.58 to –0.22, *p* < 0.001) and VR-based AVATAR therapy (SMD = –0.41, 95% CI: –0.72 to –0.09, *p* = 0.01) were associated with significant improvements in PSYRATS-AH-total scale compared to the control group (eFigure 24). No significant subgroup differences were observed between the two modalities (χ² = 0.00, *p* = 0.97). Heterogeneity across studies was low (*I*² = 0%), indicating consistent effects. For subscales, including frequency (eFigure 25) and distress (eFigure 26), computer-screen-based AVATAR therapy demonstrated significant improvement in both subscales compared to control group (frequency: SMD = –0.24, 95% CI: –0.47 to –0.02, *p* = 0.04; distress: SMD = –0.40, 95% CI: –0.56 to –0.24, *p* < 0.001). However, VR-based AVATAR therapy did not show significant improvement in both subscales compared to the control (frequency: SMD = 0.03, 95% CI: –0.29 to 0.34, *p* = 0.87; distress: SMD = –0.61, 95% CI: –1.35 to 0.13, *p* = 0.11).

### Publication bias and Leave-one-out test

For PSYRATS-AH-total scale (eFigure 27) and PSYRATS-AH-frequency (eFigure 28), visual inspection of the funnel plot showed a relatively symmetrical distribution of effect sizes around the pooled estimate, suggesting a low risk of publication bias. For PSYRATS-AH-distress (eFigure 29), visual inspection of the funnel plot revealed asymmetry, suggesting potential publication bias. One study^24^ appeared to deviate substantially from the funnel shape. Egger’s test was not performed due to the inclusion of fewer than 10 studies.

For PSYRATS-AH-total scale (eFigure 30) and PSYRATS-AH-distress (eFigure 31), excluding each study in turn yielded SMD ranging from –0.38 to –0.43 and -0.37 to −0.43, all of which remained statistically significant (*p* < 0.001). For PSYRATS-frequency (eFigure 32), when excluding one study^23^, AVATAR therapy showed a significant effect compared to the control group. However, when any of the other studies were excluded, the effect of AVATAR therapy remained statistically non-significant.

## Discussion

This study showed that AVATAR therapy was significantly associated with improvements in AH severity, especially in reducing AH-induced distress in patients with medication-resistant AH. In addition, overall psychotic symptoms and positive symptoms also demonstrated significant reductions compared to control therapies. For the secondary outcomes, AVATAR therapy was significantly associated with enhanced quality of life and reductions in depressive and anxiety symptoms. However, no significant effect was observed in altering beliefs about AH. In long-term efficacy, most therapeutic effects were sustained three months after the end of AVATAR therapy, with AH severity exhibiting a slightly further improvement. Finally, the all-cause discontinuation rate of AVATAR therapy did not differ from that of the control group.

Previous studies have shown that in patients with schizophrenia, AH severity is positively correlated with depressive and anxiety symptoms^25,26^. Moreover, psychotic symptoms, including positive and negative symptoms, as well as AH, depression, and anxiety, have been negatively associated with quality of life in this population^27–30^. These findings align with the results of our study. Importantly, most participants in the AVATAR therapy trials included in this meta-analysis experienced distressing AH despite long-term antipsychotic treatment, with approximately one-third receiving clozapine. AVATAR therapy, which targets patients’ cognitive interpretations of AH and enhances coping strategies^16^, appears to sustain its therapeutic benefits three months after discontinuing AVATAR therapy. Given its efficacy and acceptability, AVATAR therapy represents a promising intervention for individuals experiencing distressing medication-resistant AH.

Although AVATAR therapy significantly reduced AH severity, particularly in the distress domain, it did not lead to significant improvements in the beliefs about AH compared to control therapies. This finding suggests that while brief intervention therapies may enhance patients’ coping abilities with AH, they remain insufficient to induce substantial changes in underlying beliefs. Similar therapeutic effects have been observed in the application of CBT for patients with schizophrenia. A meta-analysis of 18 RCTs demonstrated that CBT had a moderate effect size (Hedge’s g = 0.44, 95% CI = 0.26 to 0.61, *I*^2^ = 0%) for hallucinations in schizophrenia. However, for delusions (another form of beliefs), the effect size was smaller, with moderate heterogeneity (Hedge’s g = 0.36, 95% CI = 0.08 to 0.63, *I*^2^ = 55.5%)^31^. Previous studies on CBT for schizophrenia have identified Insight and psychiatric symptom severity, particularly negative symptoms, as predictors of treatment outcomes^12,13^. In our analysis, AVATAR therapy was not significantly associated with improvements in negative symptoms, either.

In subgroup analysis of VR-based versus computer screen-based AVATAR therapy, the results suggest that both computer-screen-based and VR-based AVATAR therapy are effective in reducing overall auditory hallucinations, with no significant difference between modalities. However, only the computer-screen-based version showed statistically significant improvements in specific symptom dimensions of frequency and distress. Given that study protocols were highly heterogeneous in terms of study design, patient populations, and control conditions, these findings should be interpreted with caution. For instance, the most recent large-sample trial on computer-based AVATAR therapy (Garety et al.^22^) reported relatively larger statistical differences, which may be explained by the fact that their participants were not treatment-resistant, the control group received treatment as usual rather than an active control such as CBT (as in Dellazizzo et al.^21^ or Liang et al.^23^, both of which were in the VR-based group), and a substantial proportion of patients did not have a schizophrenia diagnosis, which likely improved overall prognosis. It is therefore not surprising that participants in that study responded more favorably, since AVATAR therapy was not administered as a last-resort treatment. These differences are unlikely to be attributable simply to the use of a computer screen rather than VR, and this nuance should be highlighted when interpreting results. Moreover, the sample size of the VR-based group was relatively small, approximately one-third of the computer screen-based group. In addition, when the PSYRATS scale is divided into subscales, the score ranges become narrower, making it more difficult to reach statistical significance. Notably, in the distress domain, the effect size (SMD = –0.61) was relatively large; however, the 95% confidence interval still crossed zero. In the VR-based group, the study by du Sert et al.^24^ reported a larger effect size on AH-distress symptoms compared to other trials. In addition to relatively small sample size, study design may be another key contributing factor, as this trial adopted an open-label delayed treatment design. The delayed group did not receive any other non-pharmacological interventions during the waiting period and may have even experienced a nocebo effect, potentially leading to an overestimation of the treatment effect in the AVATAR arm. Nevertheless, more future clinical trials of VR-based AVATAR therapy are needed to confirm its therapeutic effects on AH.

Regarding another non-pharmacological intervention for AH or positive symptoms for schizophrenia, transcranial direct current stimulation (tDCS) was found to improve positive symptoms (SMD = 0.17, 95% CI = 0.001 to 0.33) and AH (SMD = 0.36, 95% CI = 0.02 to 0.70) compared by sham interventions, as reported by a meta-analytic study with 16 RCTs^32^. It is important to note that although these results were statistically significant, the lower bound of the 95% confidence intervals for the effect sizes were very close to zero^32^. Another non-invasive brain stimulation method, transcranial magnetic stimulation (TMS), did not show a statistically significant effect on auditory hallucinations (AH) in schizophrenia. However, its effect on positive symptoms (measured by PANSS) was similar to that of AVATAR therapy (MD = −2.14, 95%Cl = −3.15 to −1.14)^33^.

Several trials of VR interventions have focused on social skills, social cognition, or vocational training in patients with schizophrenia, and these studies have showed promising results on negative symptoms and social function^34^. The therapeutic goals and the specific techniques or strategies employed play a crucial role in shaping the outcomes of these VR-assisted interventions for schizophrenia. For example, a VR-based trial for schizophrenia implemented a therapy approach integrating CBT and cognitive remediation, which showed significant efficacy on negative symptoms^35^. This approach incorporated activity appraisal and re-appraisal, an errorless learning environment, and structured engagement in enjoyable daily activities^35^. Additionally, motivation training was facilitated through VR-generated scenarios^35^. In contrast, AVATAR therapy primarily focuses on enabling therapists and patients to practice coping with AH within a VR setting, with less emphasis on motivation, negative symptoms, or insight. This difference in therapeutic focus may contribute to variations in treatment outcomes. However, these findings are encouraging, as they indicate that versatility of VR in addressing various symptoms of schizophrenia, enabling individualized treatment approaches. Future AVATAR therapy may benefit from aiming at enhancing insight and addressing both positive and negative symptoms to improve treatment efficacy.

Previous research suggested that higher levels of voice characterization (complex characterization) were associated with greater participant engagement in AVATAR therapy and everyday behavioral engagement with voices^36^. However, our largest included study, Garaty et al., did not support this finding through moderation analysis. Their research reported very few demographic variables moderated treatment effects. The only indication of a moderator was that an earlier onset of auditory hallucinations might be associated with larger treatment effects^22^. Our second largest included study, Craig et al., using logistic regression analysis, also reported no significant predictors^20^. Additionally, research indicates that individuals’ openness to AVATAR therapy, along with the content and usability of the avatar meeting their expectations, will influence their motivation to accept AVATAR therapy^37^. Furthermore, dyadic interactions between participants and avatars may also affect treatment outcomes, with the avatar (provocation)–patient (self-affirmation) dyad showing a positive correlation with the efficacy of psychotic symptoms^38^.

Our study has some limitations. First, in our analysis, control therapies varied across studies, including treatment as usual, supportive therapy, and CBT. However, most pooled outcome measures demonstrated low heterogeneity (*I*²), suggesting that the differences in control therapies did not substantially impact the estimated efficacy. Second, there was heterogeneity among study participants. Although the majority of participants were diagnosed with schizophrenia or schizoaffective disorder, in the largest RCT^22^, only approximately 52% of participants had these diagnoses. Third, the sample sizes of the included AVATAR RCTs remain relatively small, although the large two RCTs (with more than 150 participants)^20,22^ have reported positive results. Fourth, because of the nature of psychotherapy-related clinical trials, blinding was generally limited to single-blind or assessor-blind designs. Fifth, baseline differences in symptom severity, level of insight, and cognitive function might influence the efficacy of AVATAR therapy. However, due to the limited number of studies, meta-regression could not be performed. Sixth, in PSYRATS-AH-distress, one study^24^ showed a small-study effect, indicating potential publication bias. However, in the leave-one-out analysis, the significant effect of AVATAR therapy on PSYRATS-AH distress remained unchanged after excluding this study. Finally, AVATAR therapy is not a widely adopted or standardized treatment, and variations in therapeutic approaches among clinicians may contribute to differences in treatment effectiveness.

In conclusion, AVATAR therapy has shown certain efficacy in patients with medication-resistant AH. Compared to other non-pharmacological interventions such as CBT, tDCS, and TMS, avatar therapy has an effect size that is not inferior and may even be slightly larger. Additionally, the therapeutic effects are sustained even after the treatment sessions have concluded, and it is accepted by patients. Clinicians should consider applying this therapy on patients who do not benefit sufficiently from medication. The training of therapists for AVATAR therapy and the continuous optimization of treatment content are also directions that require future efforts.

## Supplementary information

Supplementary Data

## Data Availability

Available on reasonable request.
