# Supplementary material for: AVATAR therapy for medication-resistant auditory hallucination in patients with psychosis: a systematic review and meta-analysis
Source: Schizophrenia (Heidelb). 2025 Dec 13;12(1):1. doi: 10.1038/s41537-025-00671-5 (PMC12770313; doi:10.1038/s41537-025-00671-5)
Supplement: Supplementary file 1 — Supplementary Data [file 41537_2025_671_MOESM1_ESM.docx]

**AVATAR Therapy for Medication-Resistant Auditory Hallucination: A Systematic Review and Meta-analysis**

eTable 1. Description of included studies

efigure 1. PRISMA 2020 flow diagram

efigure 2. Risk of bias plot

efigure 3. Summary of risk of bias

efigure 4. Comparison of avatar therapy and control in Beliefs About Voices Questionnaire persecutory belief

efigure 5. Comparison of avatar therapy and control in Beliefs About Voices Questionnaire total score

efigure 6. Comparison of avatar therapy and control in Beliefs About Voices Questionnaire Malevolence

efigure 7. Comparison of avatar therapy and control in Beliefs About Voices Questionnaire Omnipotence

efigure 8. Comparison of avatar therapy and control in Beliefs About Voices Questionnaire Benevolence

efigure 9. Comparison of avatar therapy and control in all-cause discontinuation

efigure 10. The post-treatment follow-up effect of avatar therapy compared to control in PSYRATS-AH-Total

efigure 11. The post-treatment follow-up effect of avatar therapy compared to control in PSYRATS-AH-Distress

efigure 12. The post-treatment follow-up effect of avatar therapy compared to control in PSYRATS-AH-Frequency

efigure 13. The post-treatment follow-up effect of avatar therapy compared to control in Beliefs About Voices Questionnaire persecutory belief

efigure 14. The post-treatment follow-up effect of avatar therapy compared to control in Beliefs About Voices Questionnaire total score

efigure 15. The post-treatment follow-up effect of avatar therapy compared to control in Beliefs About Voices Questionnaire Malevolence

efigure 16. The post-treatment follow-up effect of avatar therapy compared to control in Beliefs About Voices Questionnaire Omnipotence

efigure 17. The post-treatment follow-up effect of avatar therapy compared to control in Beliefs About Voices Questionnaire Benevolence

efigure 18. The post-treatment follow-up effect of avatar therapy compared to control in PANSS total score

efigure 19. The post-treatment follow-up effect of avatar therapy compared to control in PANSS positive symptoms

efigure 20. The post-treatment follow-up effect of avatar therapy compared to control in PANSS negative symptoms

efigure 21. The post-treatment follow-up effect of avatar therapy compared to control in quality of life (standardized mean difference)

efigure 22. The post-treatment follow-up effect of avatar therapy compared to control in depressive symptoms (standardized mean difference)

efigure 23. The post-treatment follow-up effect of avatar therapy compared to control in anxiety symptoms (standardized mean difference)

eFigure 24. Subgroup analysis of PSYRATS-AH-Total (standardized mean difference)

eFigure 25. Subgroup analysis of PSYRATS-AH-Frequency (standardized mean difference)

eFigure 26. Subgroup analysis of PSYRATS-AH-Distress (standardized mean difference)

eFigure 27. Funnel Plot of AVATAR therapy in PSYRATS-AH-Total (standardized mean difference)

eFigure 28. Funnel Plot of AVATAR therapy in PSYRATS-AH-Frequency (standardized mean difference)

eFigure 29. Funnel Plot of AVATAR therapy in PSYRATS-AH-Distress (standardized mean difference)

eFigure 30. Leave-one-out analysis of AVATAR therapy in PSYRATS-AH-Total (standardized mean difference)

eFigure 31. Leave-one-out analysis of AVATAR therapy in PSYRATS-AH-Distress (standardized mean difference)

eFigure 32. Leave-one-out analysis of AVATAR therapy in PSYRATS-AH-Frequency (standardized mean difference)

Appendix 1. PRISMA

Appendix 2. Complete search strategies

Appendix 3. Reasons for exclusion

eTable 1. Description of included studies

| Study  / Country  / Study design | Interventions | n/ n of male/ mean age (year) | Total sessions/ Treatment duration | Diagnoses of participants | Diagnostic criteria | Treatment-resistant definition level | % of participants treated with clozapine |
| --- | --- | --- | --- | --- | --- | --- | --- |
| Leff 2013  / UK/ assessor-blinded crossover | AVATAR | 26/ 16/ Na | 6/ 7 weeks | Schizophrenia | Clinical diagnosis | 1 | Na |
|  | TAU |  | Na/ 7 weeks |  |  |  |  |
| Craig 2018  /UK/ assessor-blinded | AVATAR | 75/ 57/ 42.5 | 6/ 12 weeks | Schizophrenia (77%)  Schizoaffective disorder (11%)  Bipolar disorder (5%)  Unspecific psychosis (5%)  Depression with psychotic symptoms (3%) | ICD-10 | 1 | About 33% |
|  | Supportive therapy | 75/ 45/ 42.9 |  |  |  |  |  |
| Du Sert 2018  /Canada/ OL-D | AVATAR | 15/ 10/ 42.9 | 6/ 7 weeks | Schizophrenia (80%)  Schizoaffective disorder (20%) | DSM-5 | 2 | 53.3% |
|  | TAU |  | Na/ 7 weeks |  |  |  |  |
| Dellazizzo 2021  / Canada/ OL | AVATAR | 37/ 29/ 43.6 | 9/ 9 weeks | Schizophrenia (77%)  Schizoaffective disorder (23%) | Clinical diagnosis | 2 | 52.1% |
|  | CBT | 37/ 27/ 41.4 |  |  |  |  |  |
| Liang 2022  / China/ OL | AVATAR | 32/ 14/ 25.3 | 6/ 7-9 weeks | Schizophrenia | Clinical diagnosis | 2 | 43.1% |
|  | CBT | 33/ 17/ 26.5 |  |  |  |  |  |
| Garaty 2024  /UK  / assessor-blinded | AVATAR-EX | 114/ 71/ 40.8 | 12/ 16 weeks | Schizophrenia (43.8%)  Schizoaffective disorder (7.8%)  Bipolar disorder (2.3%)  Unspecific psychosis (36.5%)  Depression with psychotic symptoms (9.6%) | ICD-10 | 1 | About 25% |
|  | AVATAR-BR | 116/ 72/ 39.4 | 6/ 16 weeks |  |  |  |  |
|  | TAU | 115/ 79/ 38.7 | Na/ 16 weeks |  |  |  |  |

OL-D, open-label delayed; OL, open-label; AVATAR, Audio Visual Assisted Therapy Aid for Refractory auditory hallucinations; TAU, Treatment as usual; CBT, Cognitive behavioral therapy; EX, Extend; BR, Brief; Na, not applicable; DSM-5, Diagnostic and Statistical Manual of Mental Disorders, Fifth Edition; ICD-10, International Classification of Diseases, Tenth Revision

Treatment resistant definition level 1: Persistence of psychotic symptoms, implying that the participants received some antipsychotics, without additional details.

Treatment resistant definition level 2: Failure of two or more antipsychotic treatments

efigure 1. PRISMA 2020 flow diagram


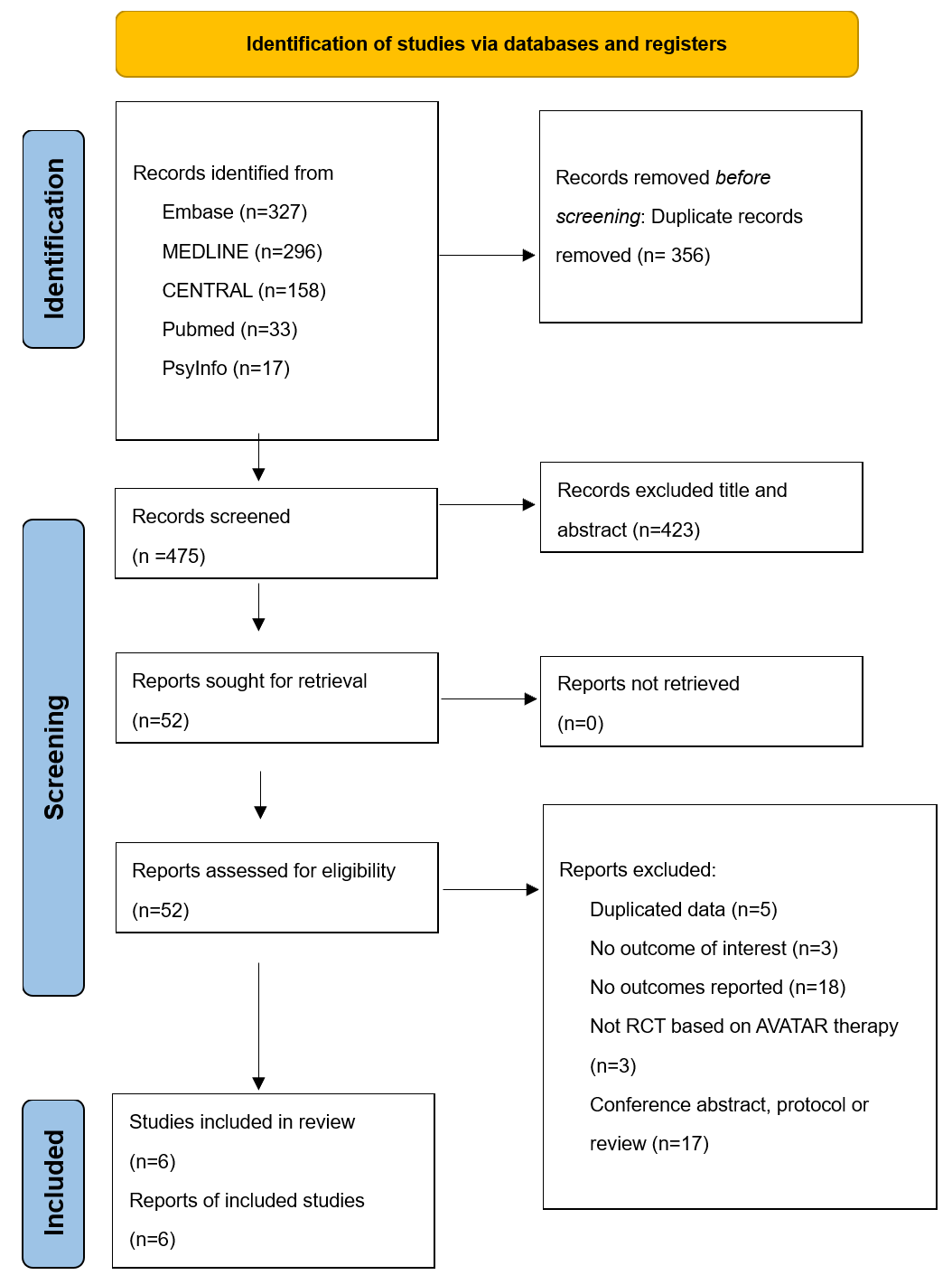


*From:*  Page MJ, McKenzie JE, Bossuyt PM, Boutron I, Hoffmann TC, Mulrow CD, et al. The PRISMA 2020 statement: an updated guideline for reporting systematic reviews. BMJ 2021;372:n71. doi: 10.1136/bmj.

efigure 2. Risk of bias plot


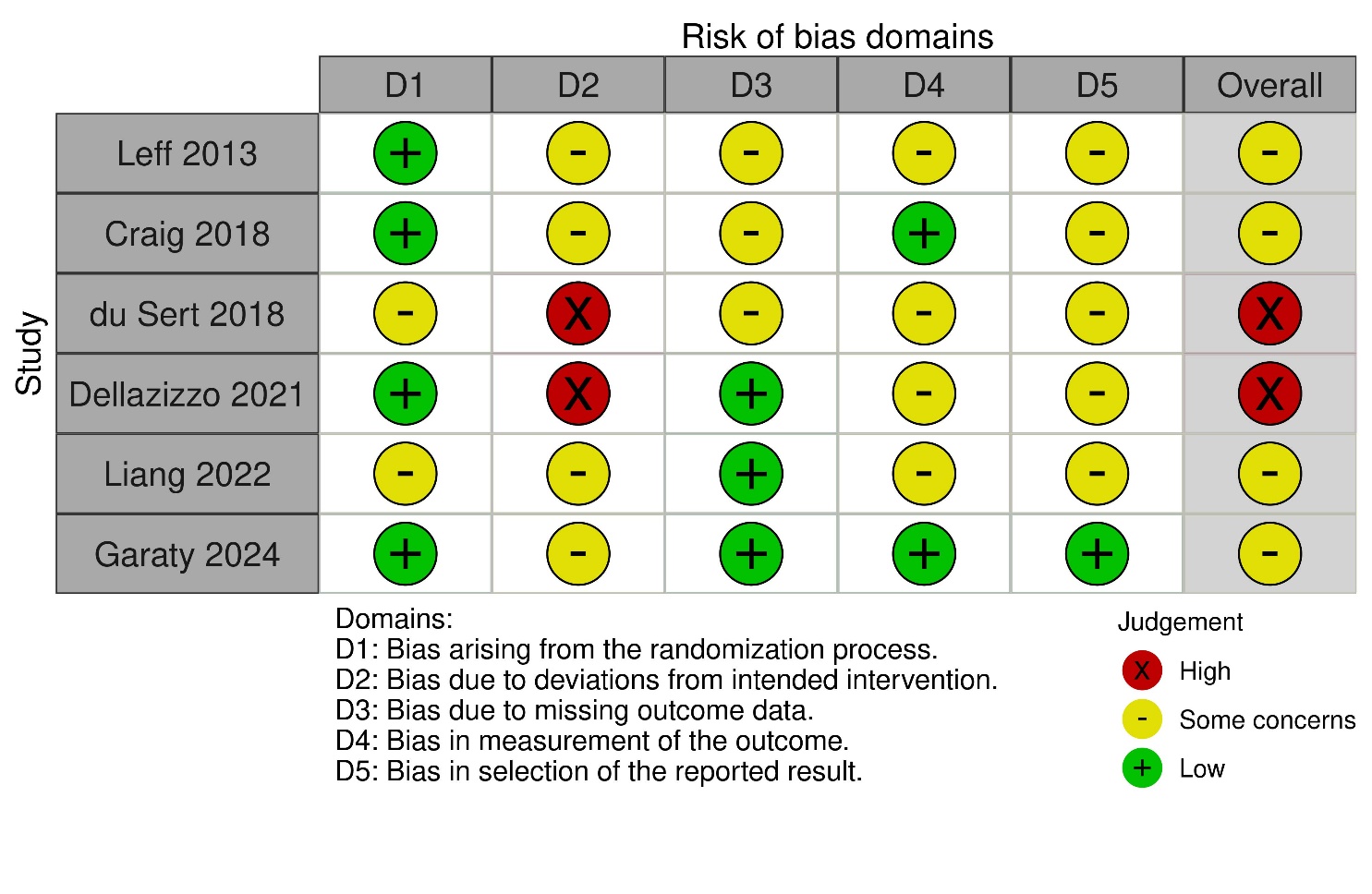


efigure 3. Summary of risk of bias


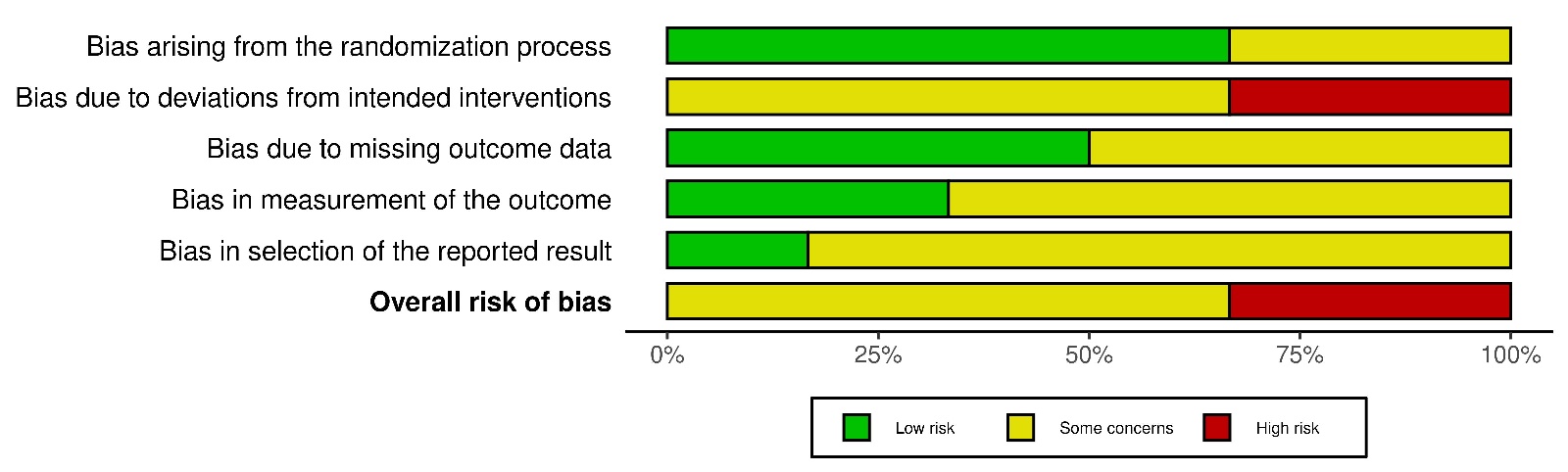


efigure 4. Comparison of avatar therapy and control in Beliefs About Voices Questionnaire persecutory belief


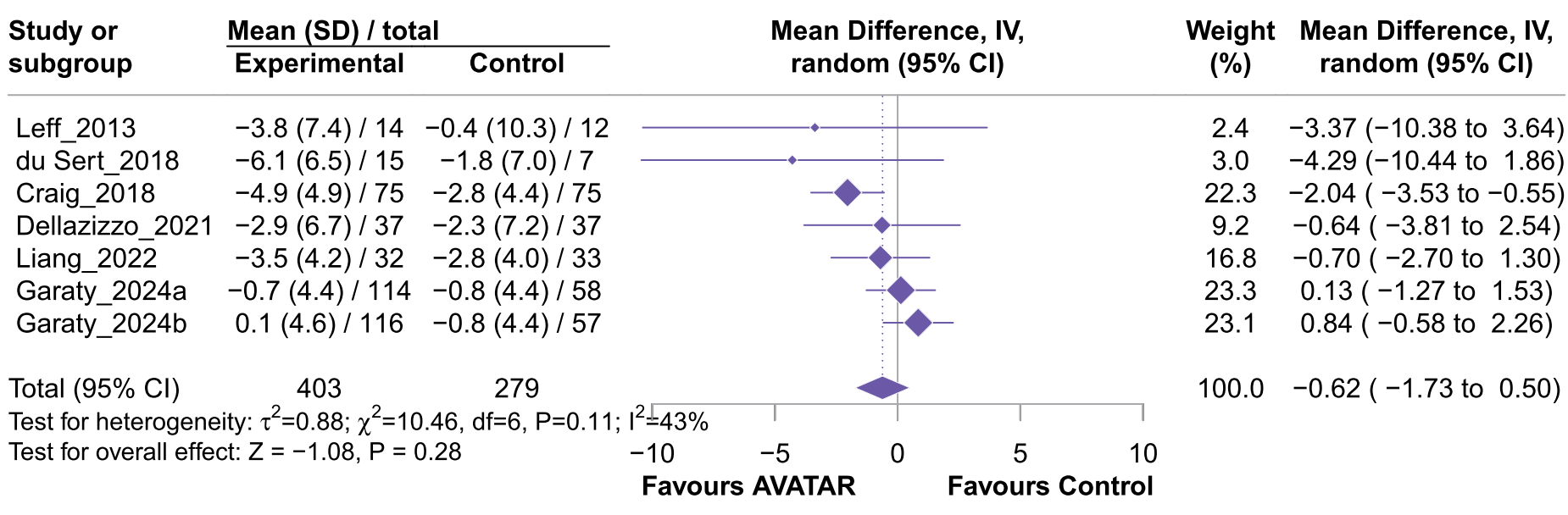


Abbreviations: CI = confidence interval; IV = inverse variance; SD = standard deviation

efigure 5. Comparison of avatar therapy and control in Beliefs About Voices Questionnaire total score


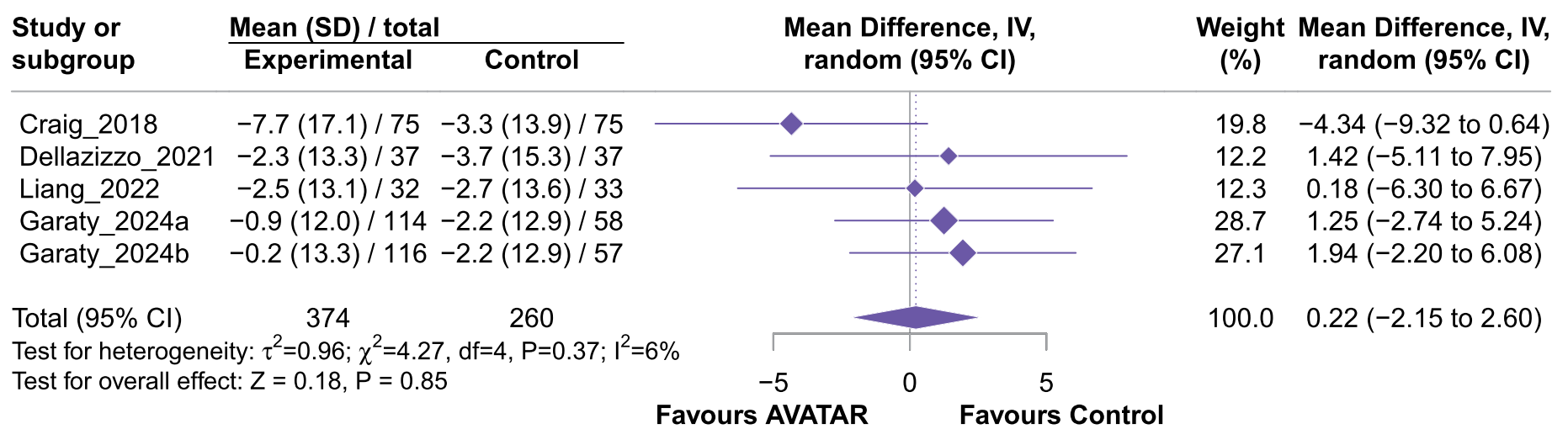


Abbreviations: CI = confidence interval; IV = inverse variance; SD = standard deviation

efigure 6. Comparison of avatar therapy and control in Beliefs About Voices Questionnaire Malevolence


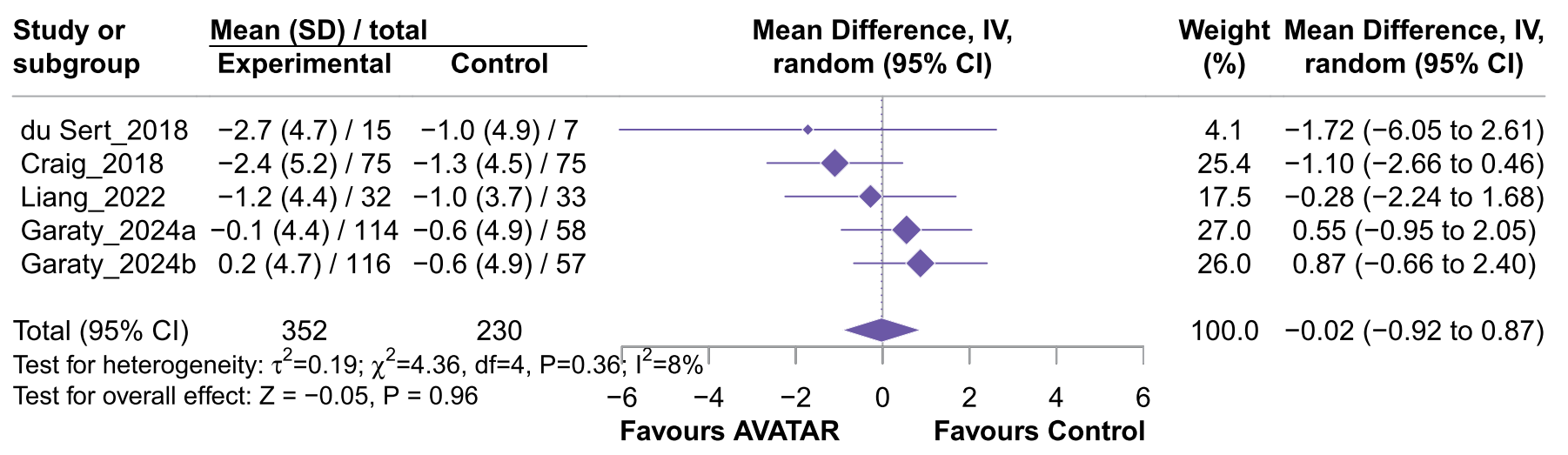


Abbreviations: CI = confidence interval; IV = inverse variance; SD = standard deviation

efigure 7. Comparison of avatar therapy and control in Beliefs About Voices Questionnaire Omnipotence


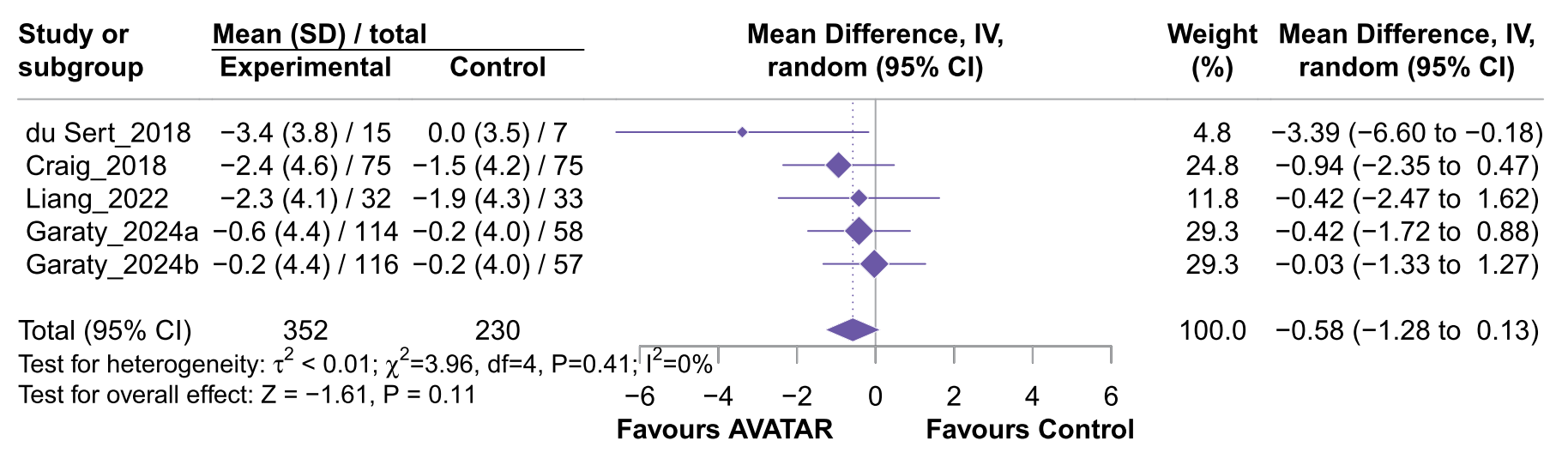


Abbreviations: CI = confidence interval; IV = inverse variance; SD = standard deviation

efigure 8. Comparison of avatar therapy and control in Beliefs About Voices Questionnaire Benevolence


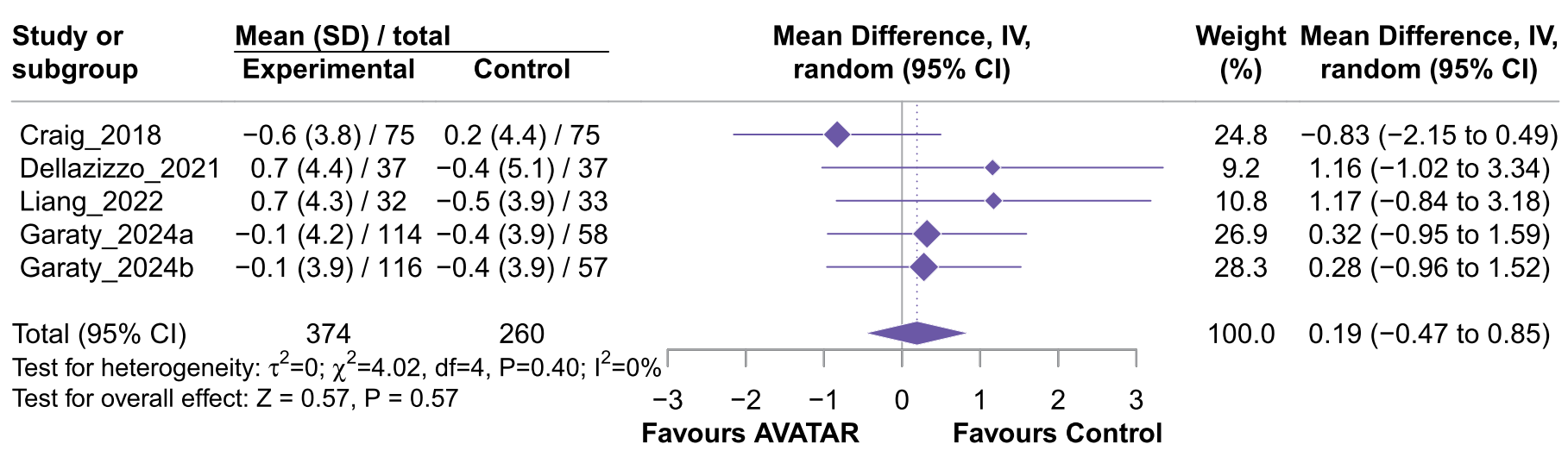


Abbreviations: CI = confidence interval; IV = inverse variance; SD = standard deviation

efigure 9. Comparison of avatar therapy and control in all-cause discontinuation


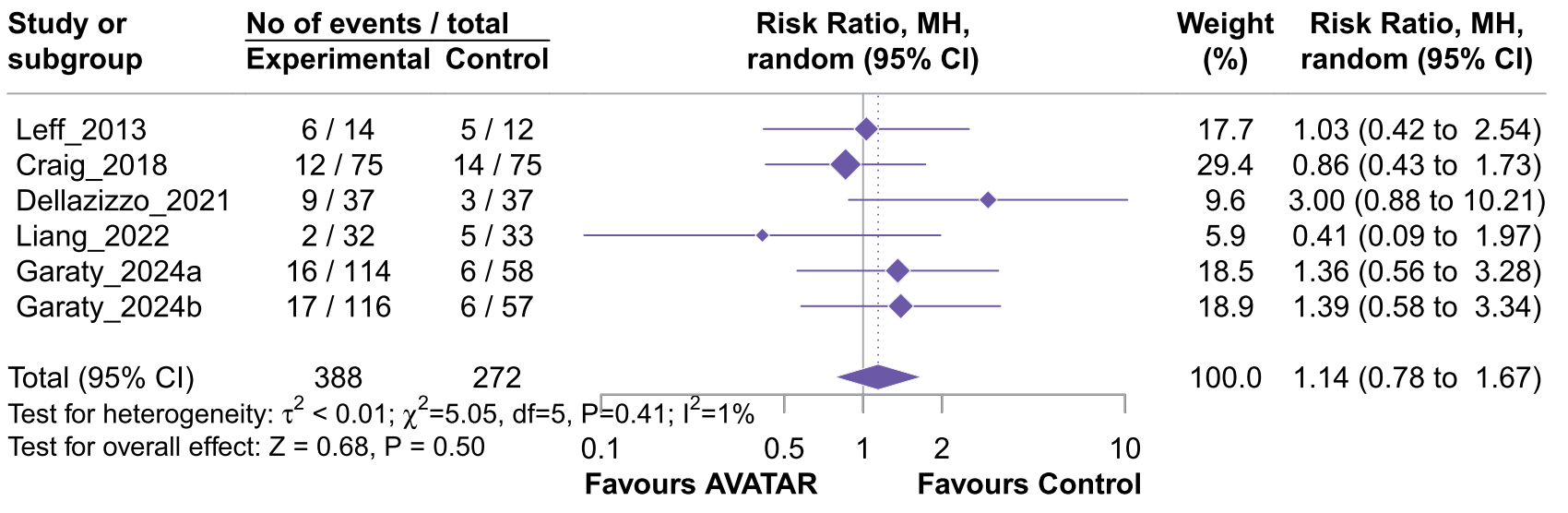
Abbreviations: CI = confidence interval; IV = inverse variance; SD = standard deviation

efigure 10. The post-treatment follow-up effect of avatar therapy compared to control in PSYRATS-AH-Total


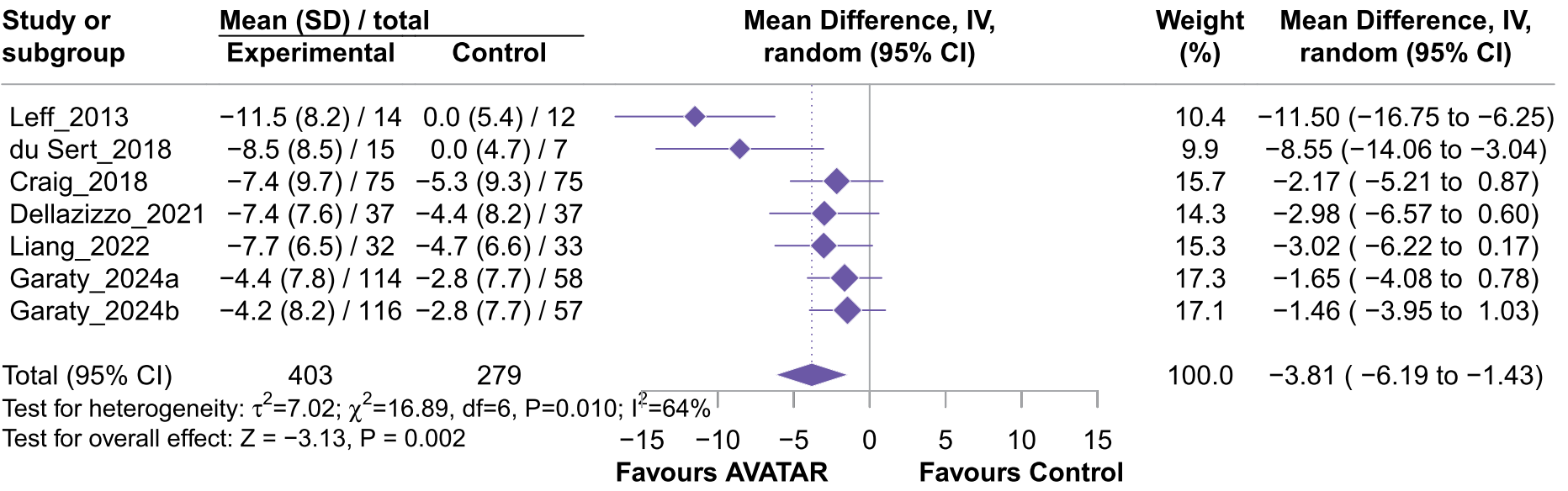
Abbreviations: CI = confidence interval; IV = inverse variance; PSYRATS-AH-Total = The Psychotic Symptom Rating Scale-Auditory Hallucination-Total Score; SD = standard deviation

efigure 11. The post-treatment follow-up effect of avatar therapy compared to control in PSYRATS-AH-Distress


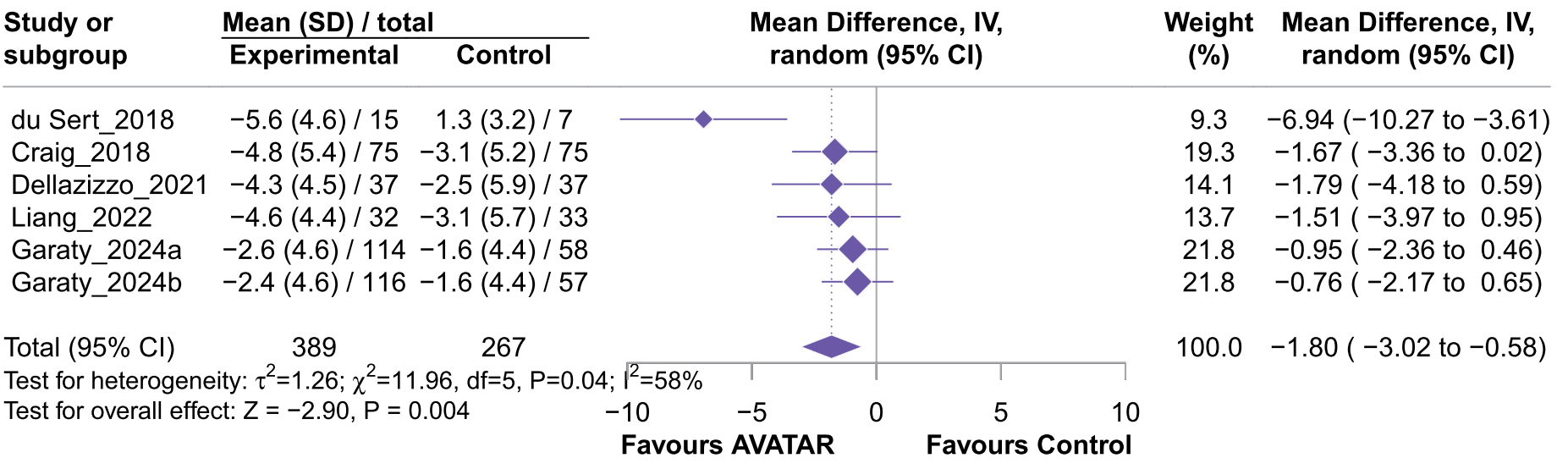
Abbreviations: CI = confidence interval; IV = inverse variance; PSYRATS-AH-D = The Psychotic Symptom Rating Scale-Auditory Hallucination-Distress; SD = standard deviation

efigure 12. The post-treatment follow-up effect of avatar therapy compared to control in PSYRATS-AH-Frequency


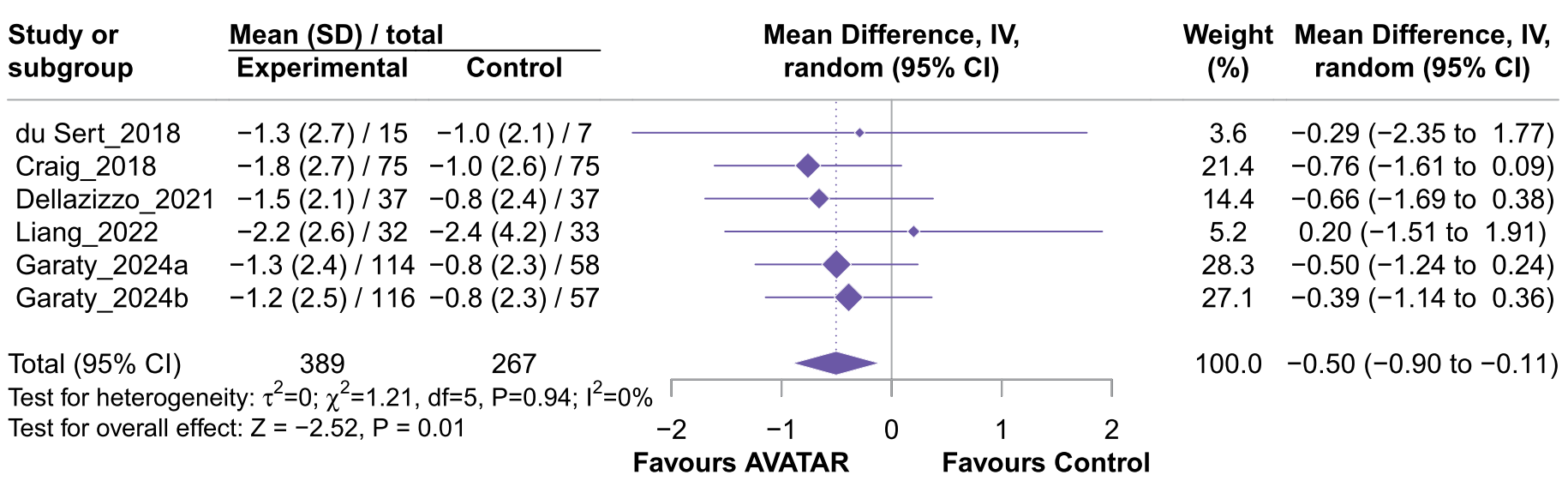
Abbreviations: CI = confidence interval; IV = inverse variance; PSYRATS-AH-F = The Psychotic Symptom Rating Scale-Auditory Hallucination-Frequency; SD = standard deviation

efigure 13. The post-treatment follow-up effect of avatar therapy compared to control in Beliefs About Voices Questionnaire persecutory belief


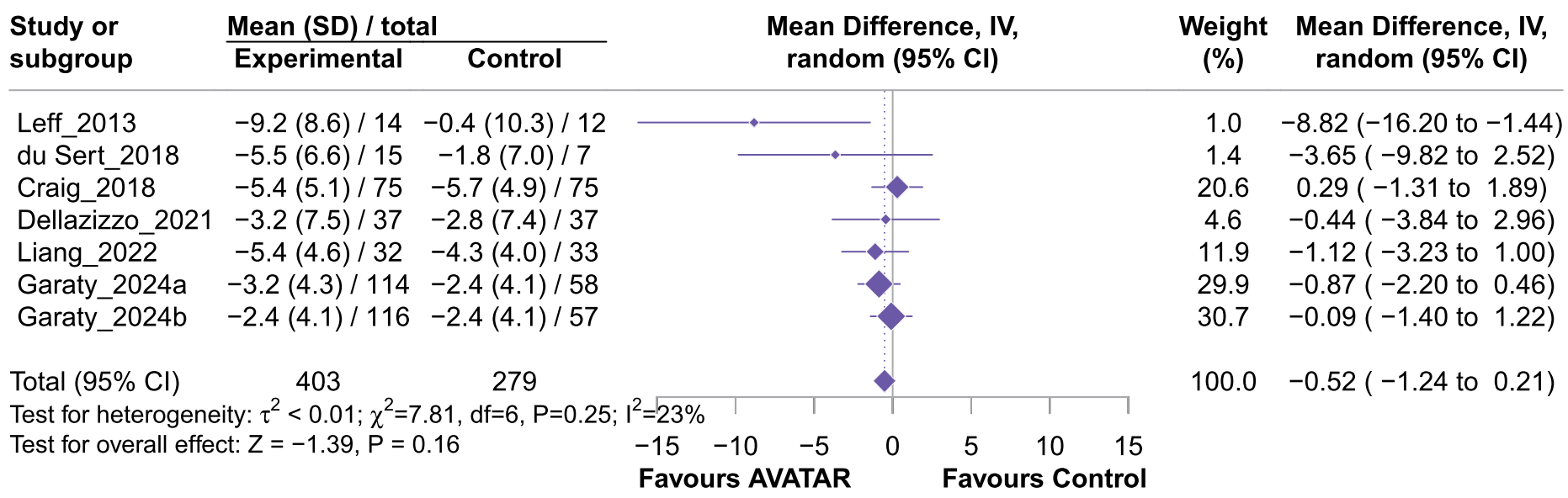
Abbreviations: CI = confidence interval; IV = inverse variance; SD = standard deviation

efigure 14. The post-treatment follow-up effect of avatar therapy compared to control in Beliefs About Voices Questionnaire total score


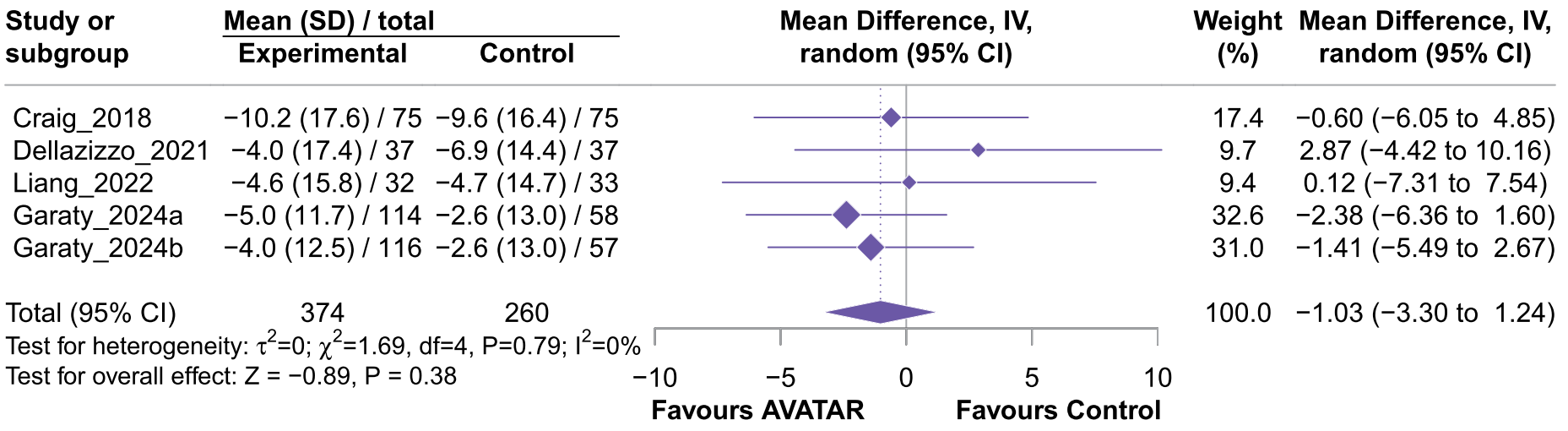


Abbreviations: CI = confidence interval; IV = inverse variance; SD = standard deviation

efigure 15. The post-treatment follow-up effect of avatar therapy compared to control in Beliefs About Voices Questionnaire Malevolence


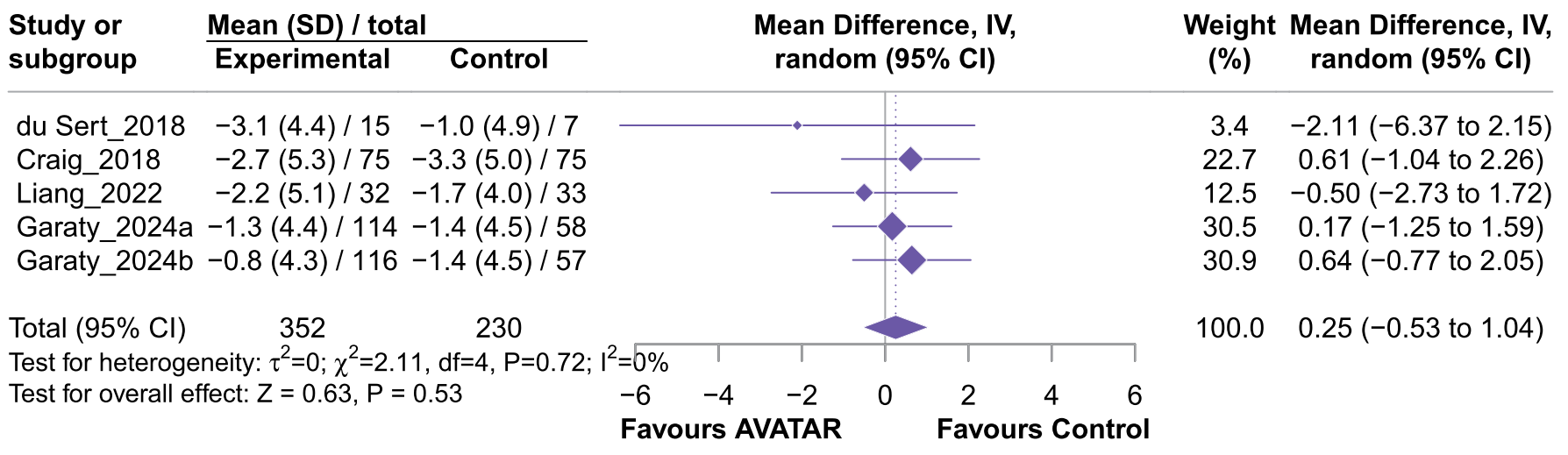


Abbreviations: CI = confidence interval; IV = inverse variance; SD = standard deviation

efigure 16. The post-treatment follow-up effect of avatar therapy compared to control in Beliefs About Voices Questionnaire Omnipotence


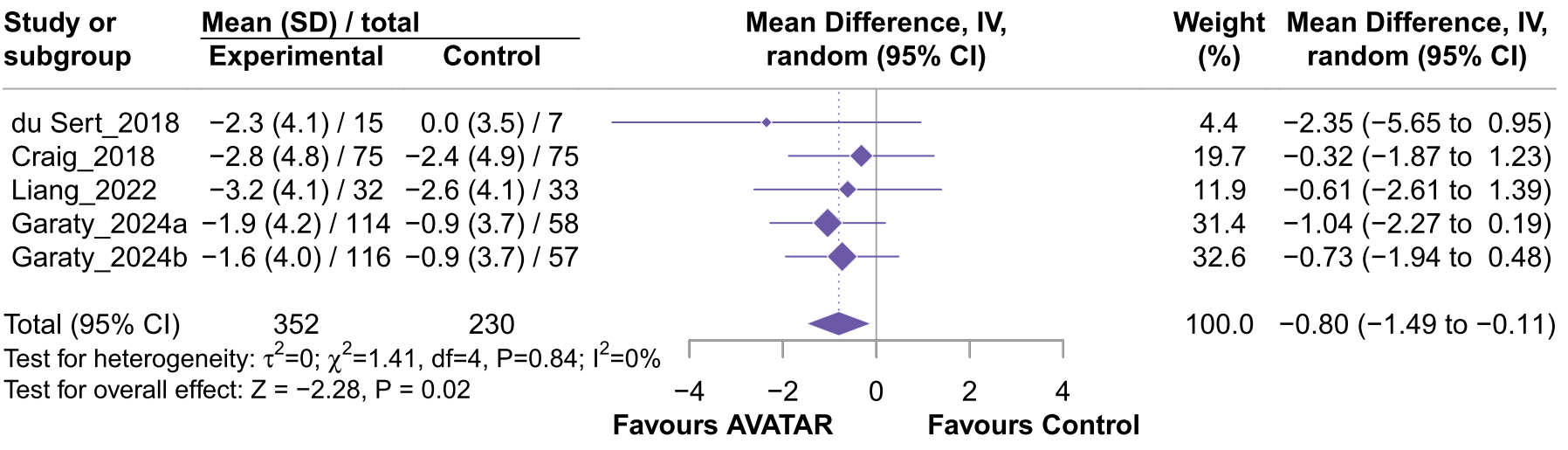


Abbreviations: CI = confidence interval; IV = inverse variance; SD = standard deviation

efigure 17. The post-treatment follow-up effect of avatar therapy compared to control in Beliefs About Voices Questionnaire Benevolence


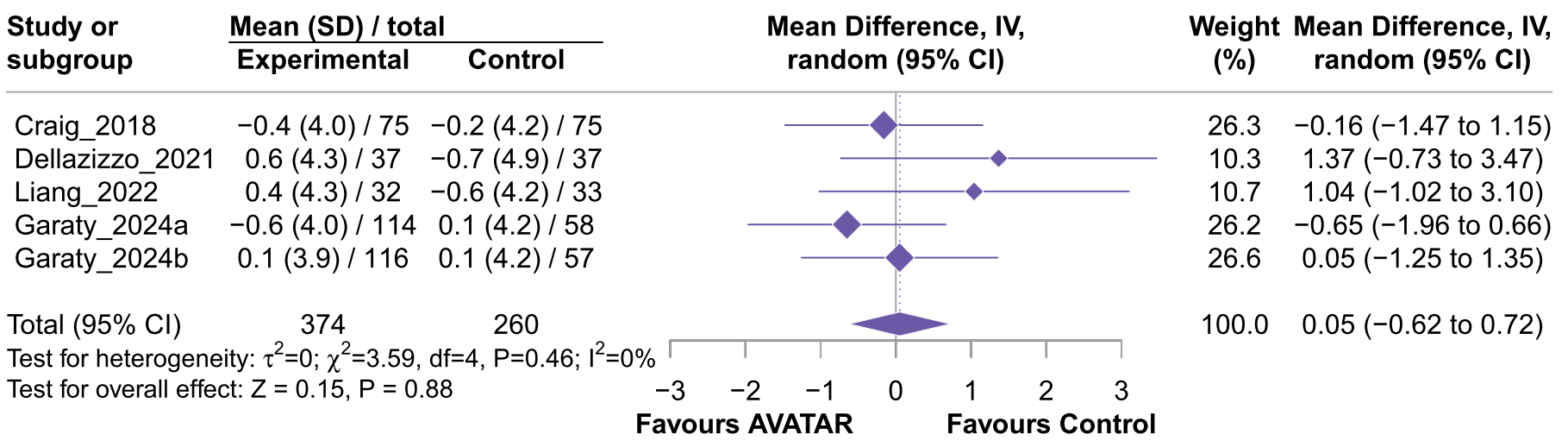


Abbreviations: CI = confidence interval; IV = inverse variance; SD = standard deviation

efigure 18. The post-treatment follow-up effect of avatar therapy compared to control in PANSS total score


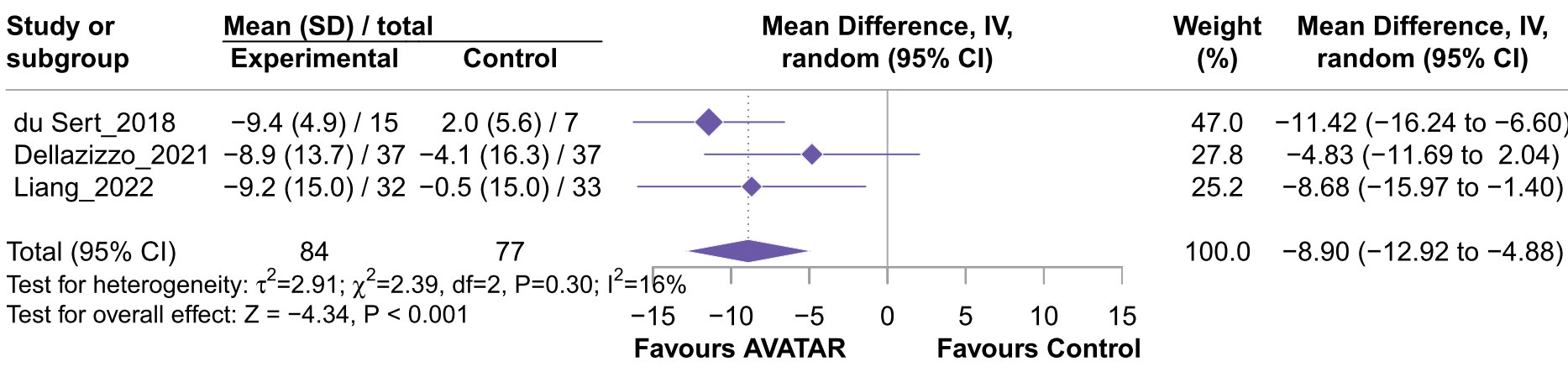


Abbreviations: CI = confidence interval; IV = inverse variance; PANSS = Positive And Negative Syndrome Scale; SD = standard deviation

efigure 19. The post-treatment follow-up effect of avatar therapy compared to control in PANSS positive symptoms


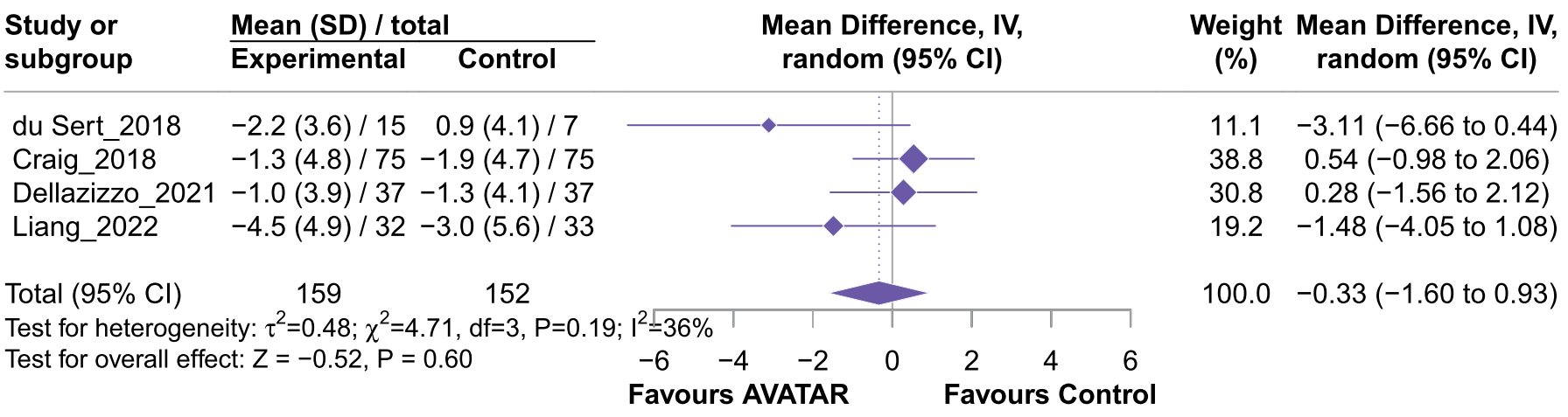


Abbreviations: CI = confidence interval; IV = inverse variance; PANSS = Positive And Negative Syndrome Scale; SD = standard deviation

efigure 20. The post-treatment follow-up effect of avatar therapy compared to control in PANSS negative symptoms


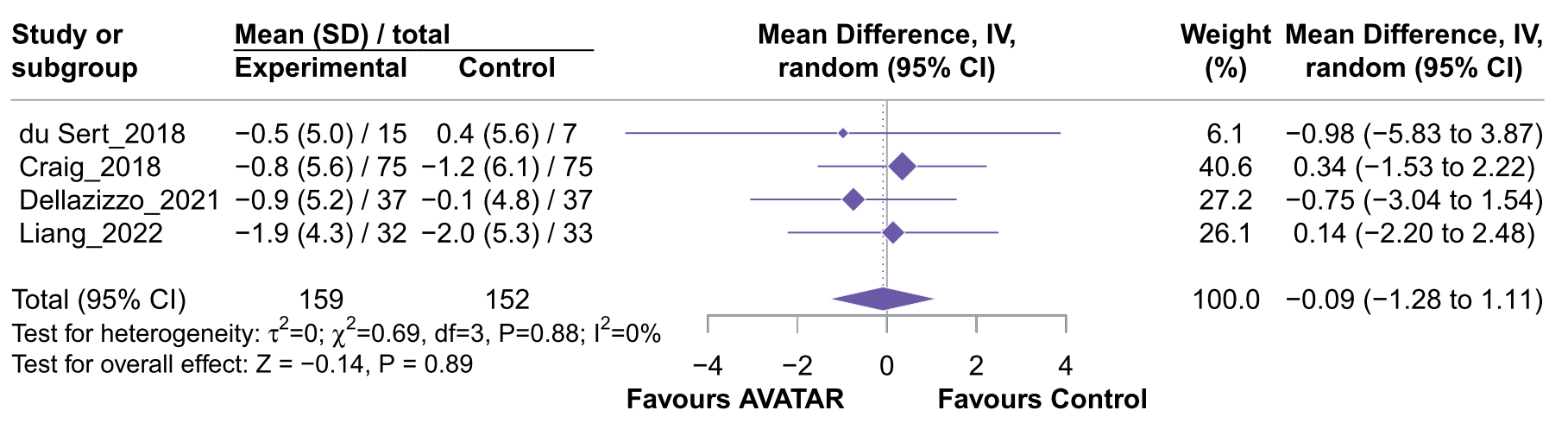
Abbreviations: CI = confidence interval; IV = inverse variance; PANSS = Positive And Negative Syndrome Scale; SD = standard deviation

efigure 21. The post-treatment follow-up effect of avatar therapy compared to control in quality of life (standardized mean difference)


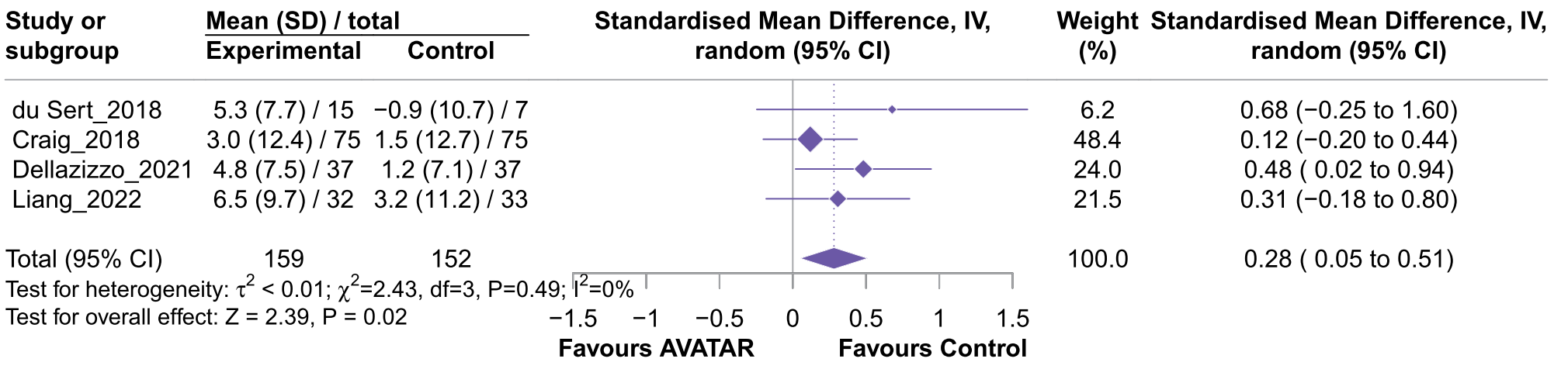


Abbreviations: CI = confidence interval; IV = inverse variance; SD = standard deviation

efigure 22. The post-treatment follow-up effect of avatar therapy compared to control in depressive symptoms (standardized mean difference)


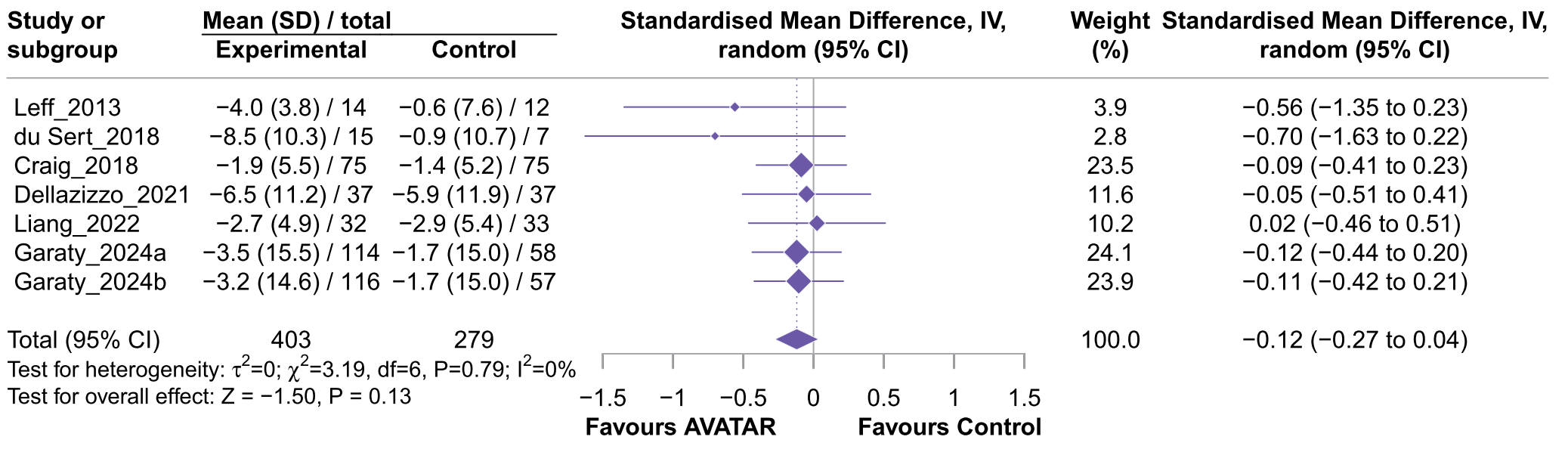


Abbreviations: CI = confidence interval; IV = inverse variance; SD = standard deviation

efigure 23. The post-treatment follow-up effect of avatar therapy compared to control in anxiety symptoms (standardized mean difference)


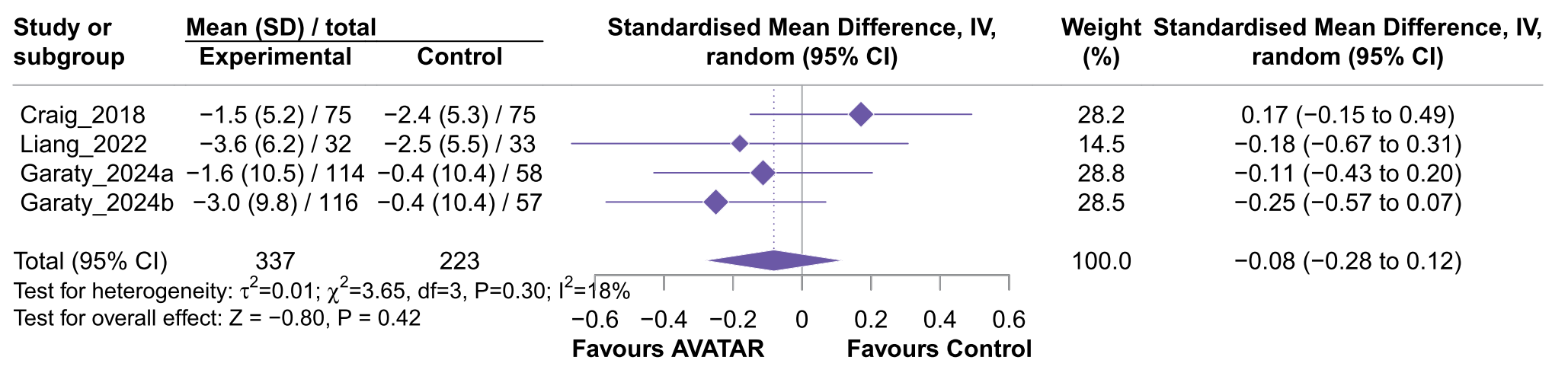


Abbreviations: CI = confidence interval; IV = inverse variance; SD = standard deviation

eFigure 24. Subgroup analysis of PSYRATS-AH-Total (standardized mean difference)


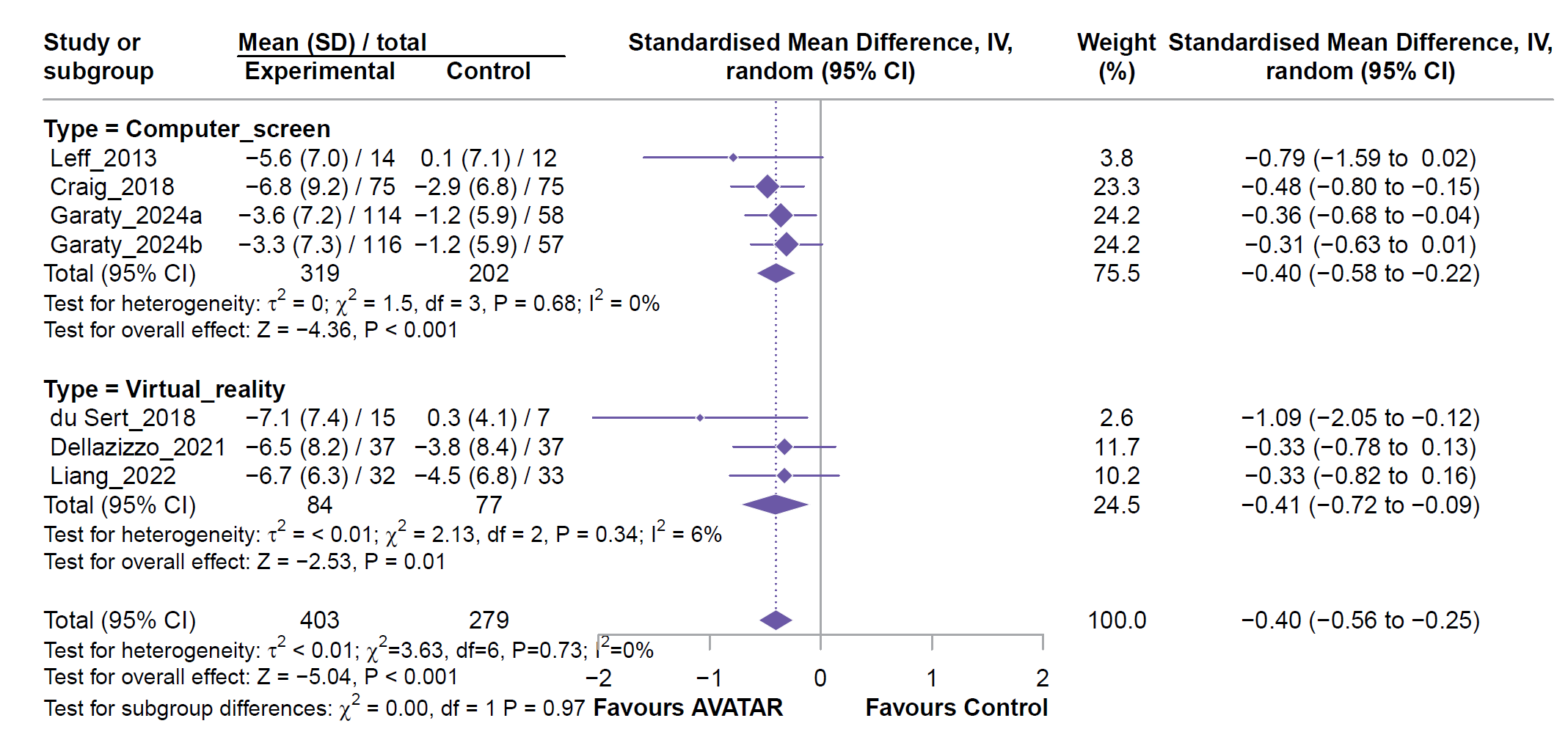


eFigure 25. Subgroup analysis of PSYRATS-AH-Frequency (standardized mean difference)


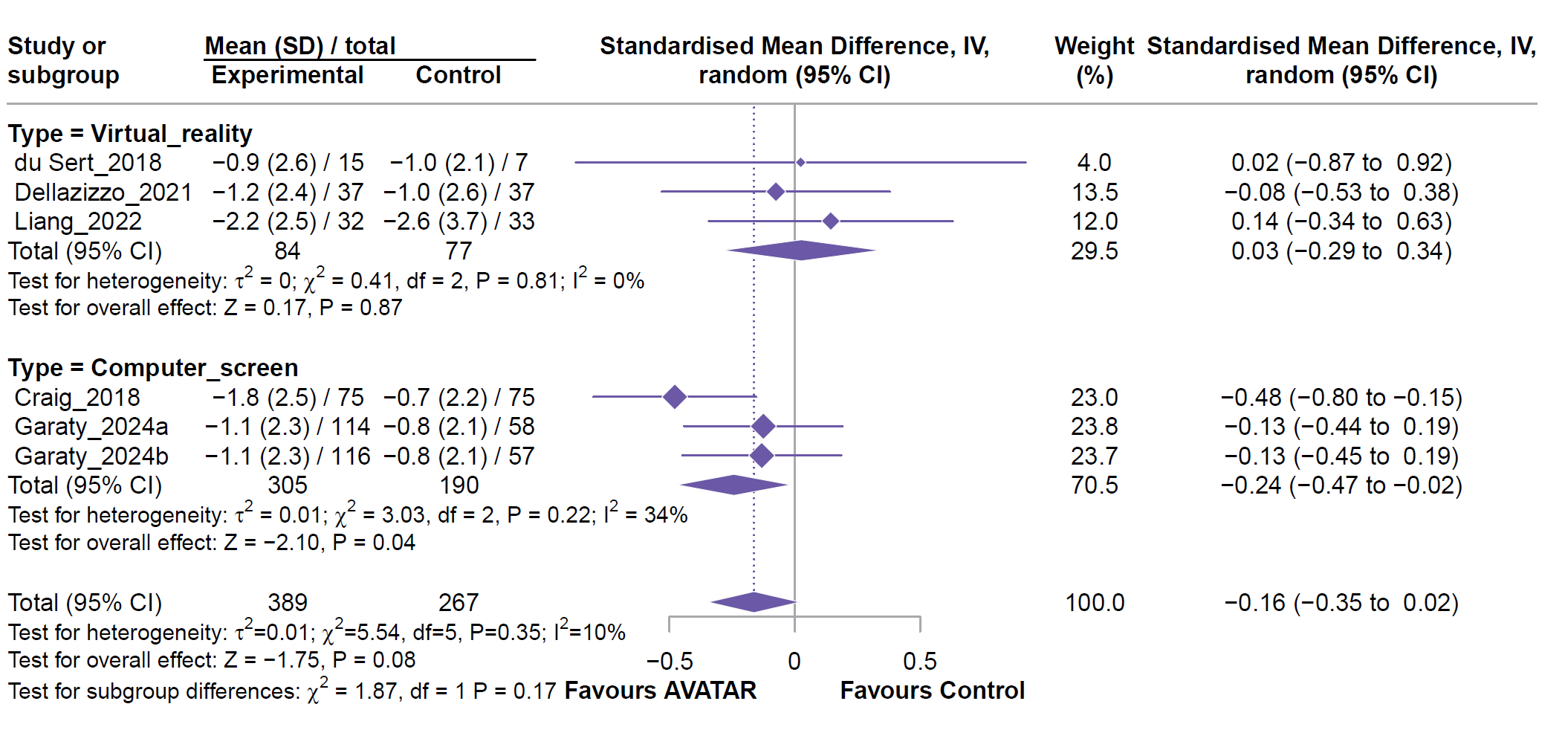


eFigure 26. Subgroup analysis of PSYRATS-AH-Frequency (standardized mean difference)


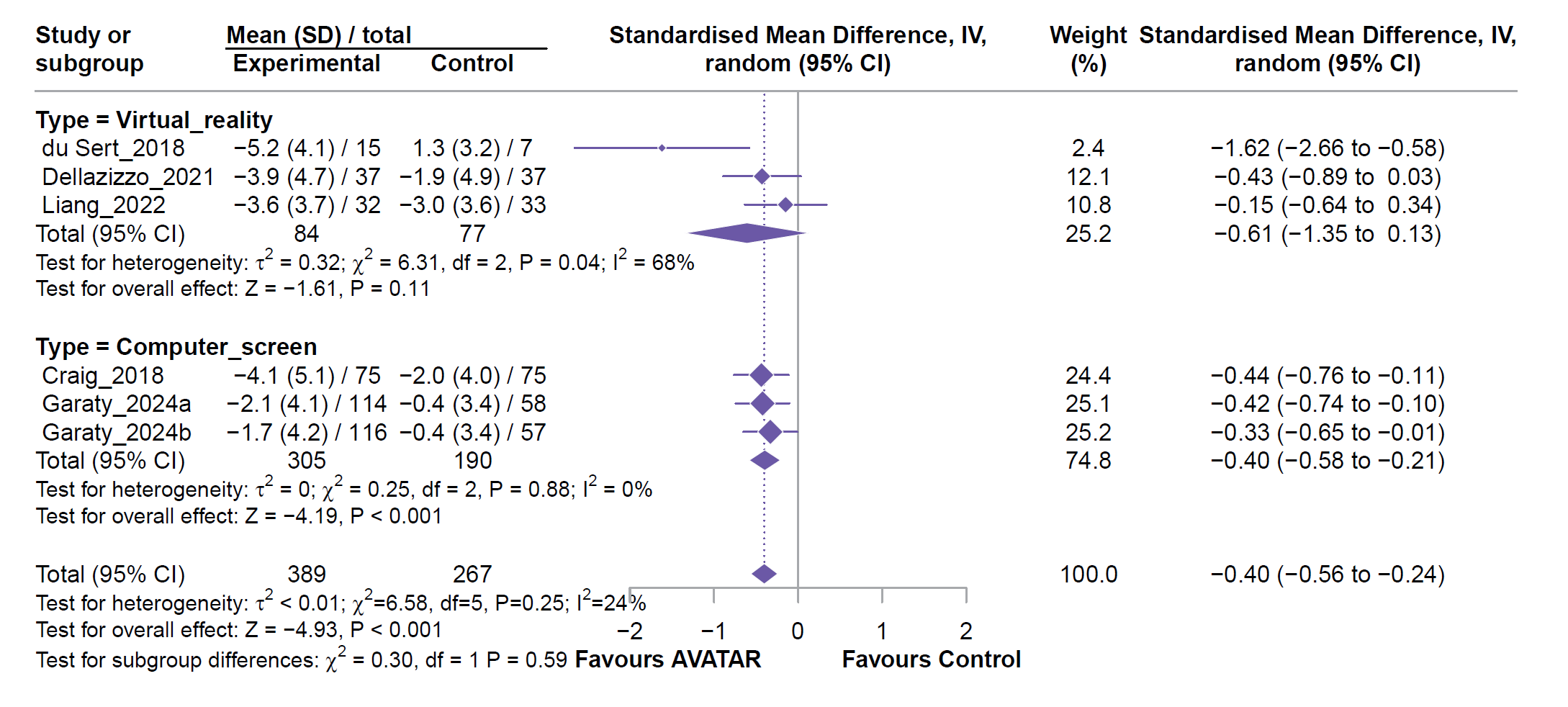


eFigure 27. Funnel Plot of AVATAR therapy in PSYRATS-AH-Total (standardized mean difference)


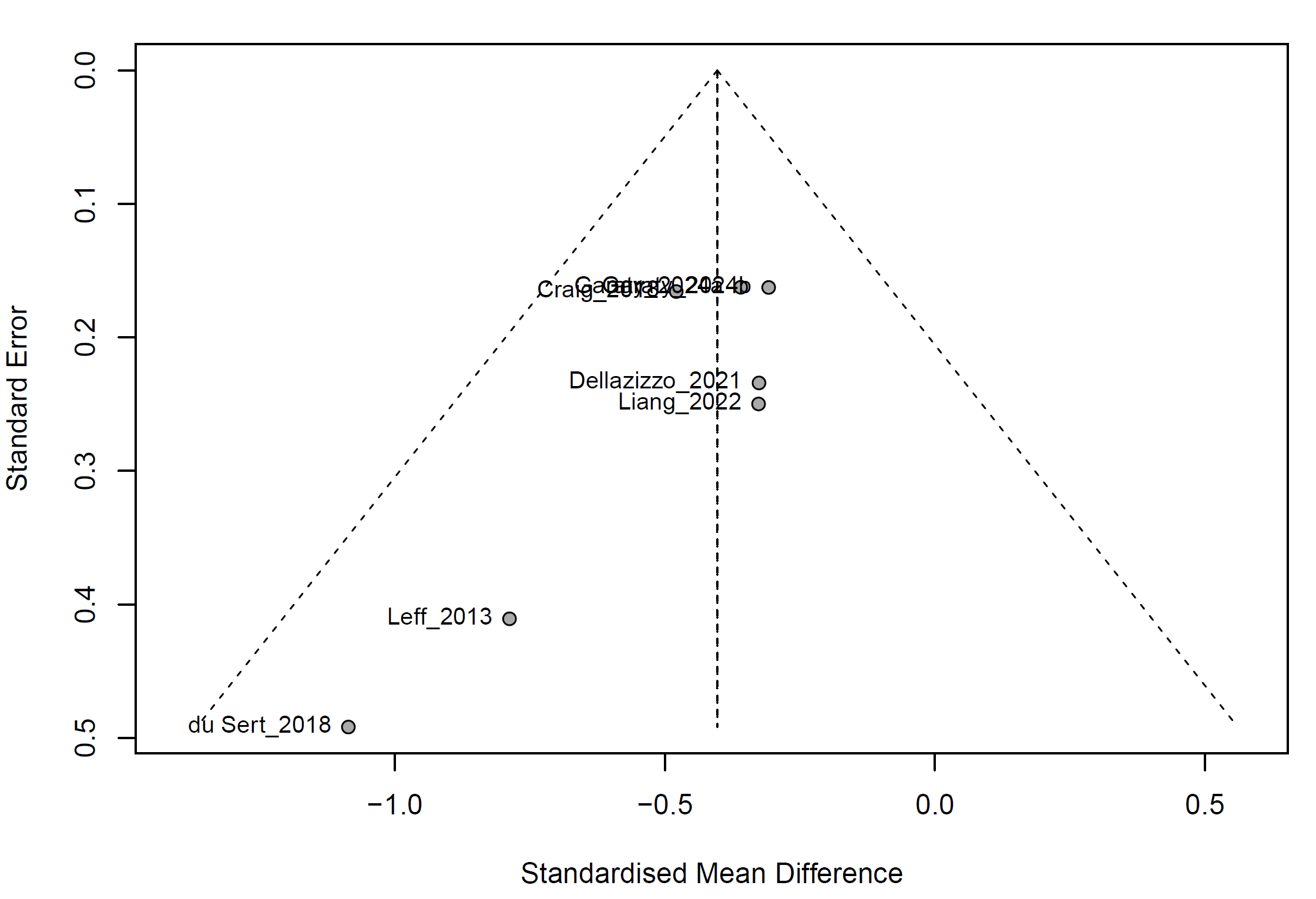


eFigure 28. Funnel Plot of AVATAR therapy in PSYRATS-AH-Frequency (standardized mean difference)


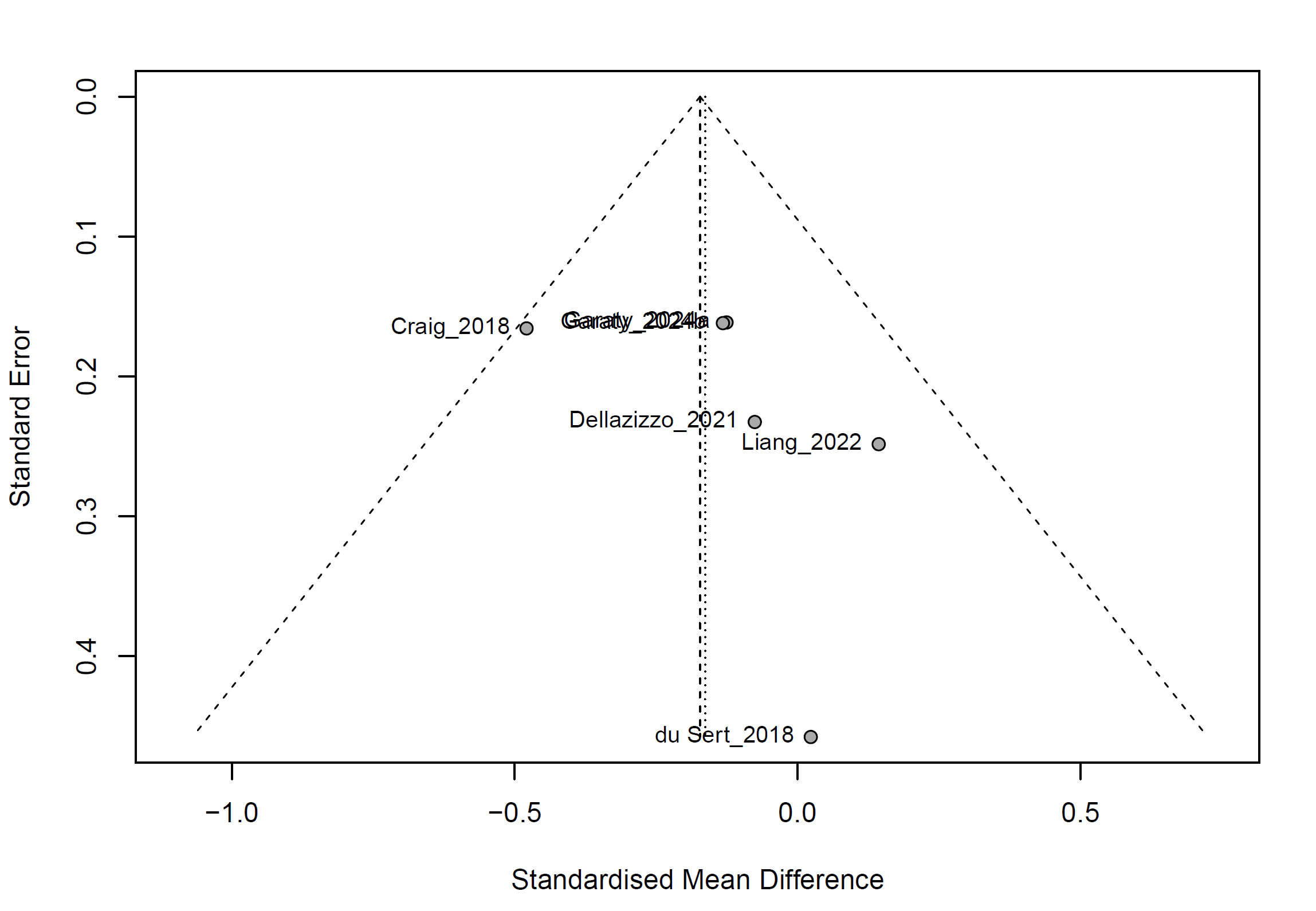


eFigure 29. Funnel Plot of AVATAR therapy in PSYRATS-AH-Distress (standardized mean difference)


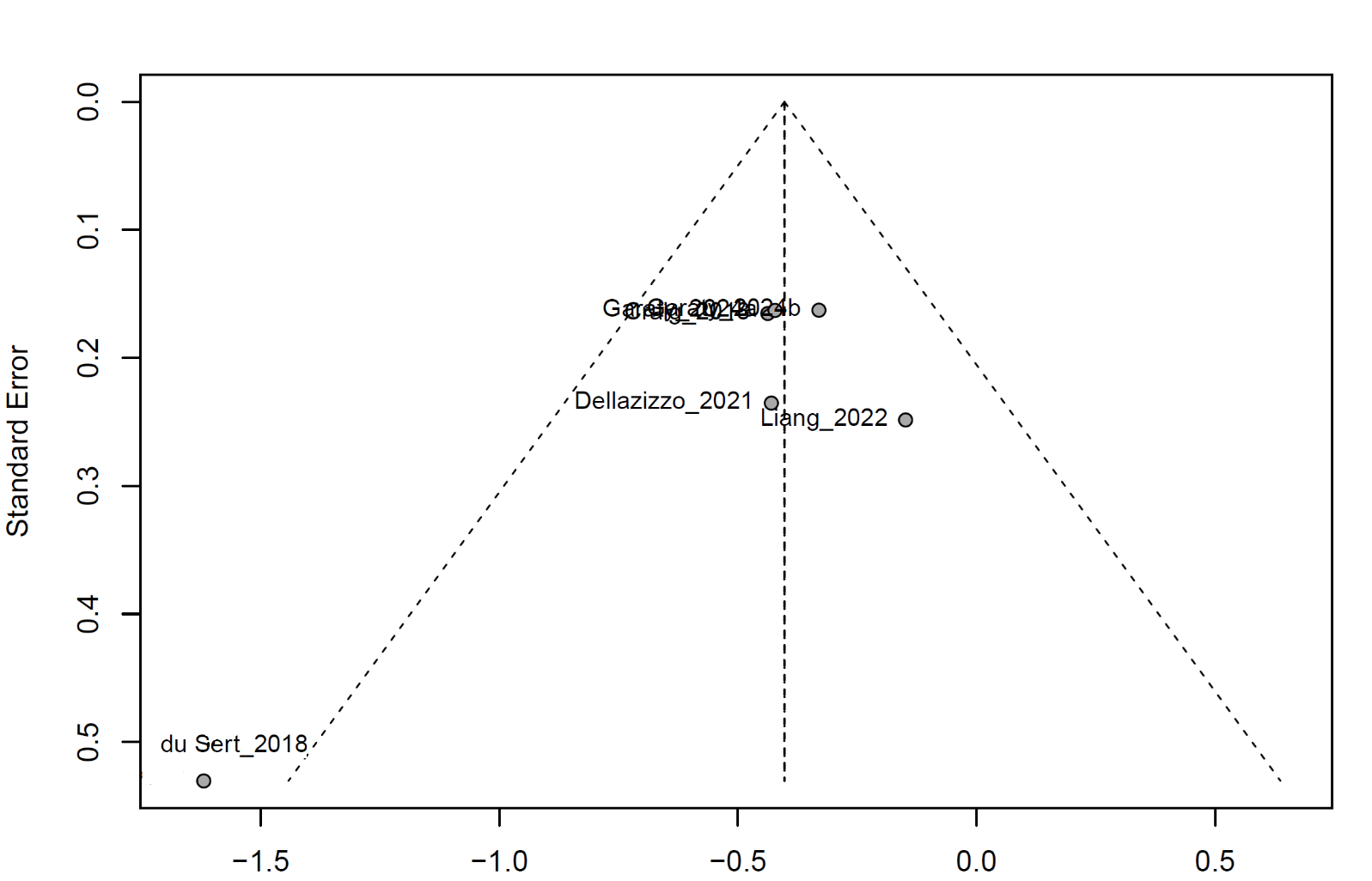


eFigure 30. Leave-one-out analysis of AVATAR therapy in PSYRATS-AH-Total (standardized mean difference)


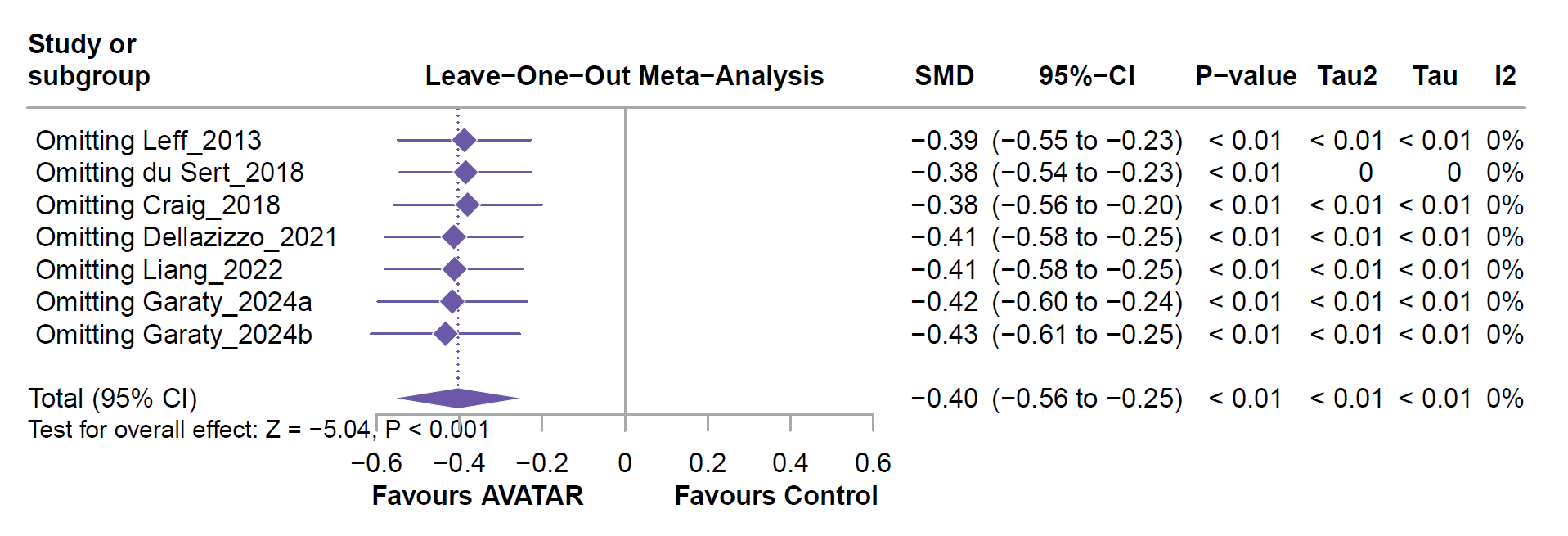


eFigure 31. Leave-one-out analysis of AVATAR therapy in PSYRATS-AH-Distress (standardized mean difference)


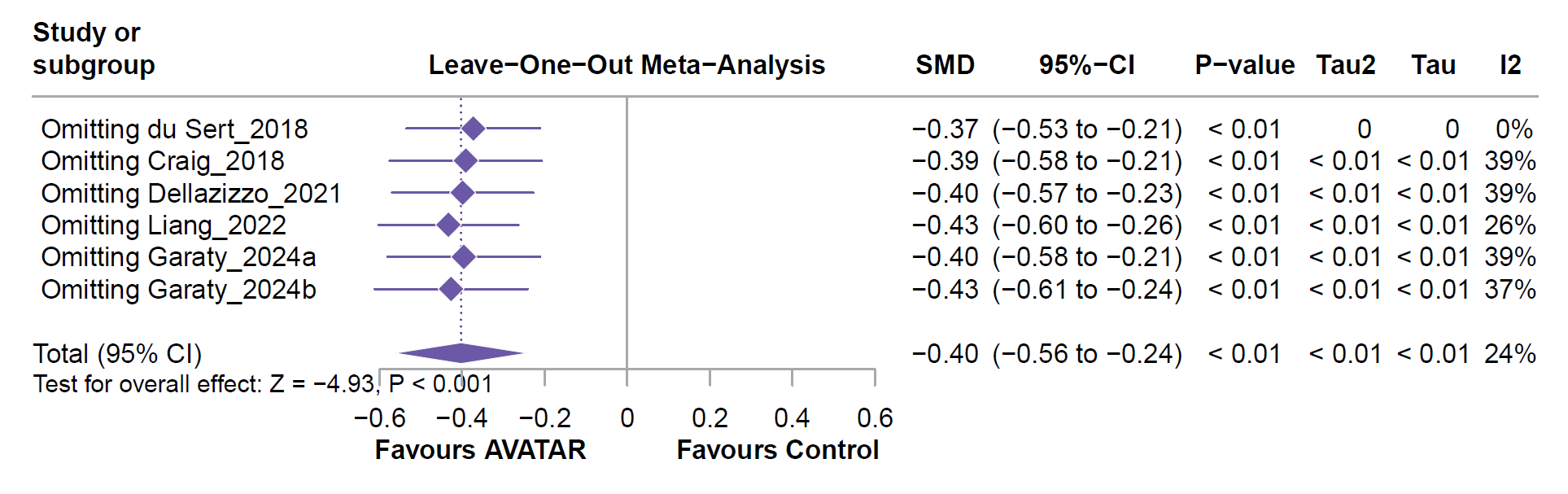


eFigure 32. Leave-one-out analysis of AVATAR therapy in PSYRATS-AH-Frequency (standardized mean difference)


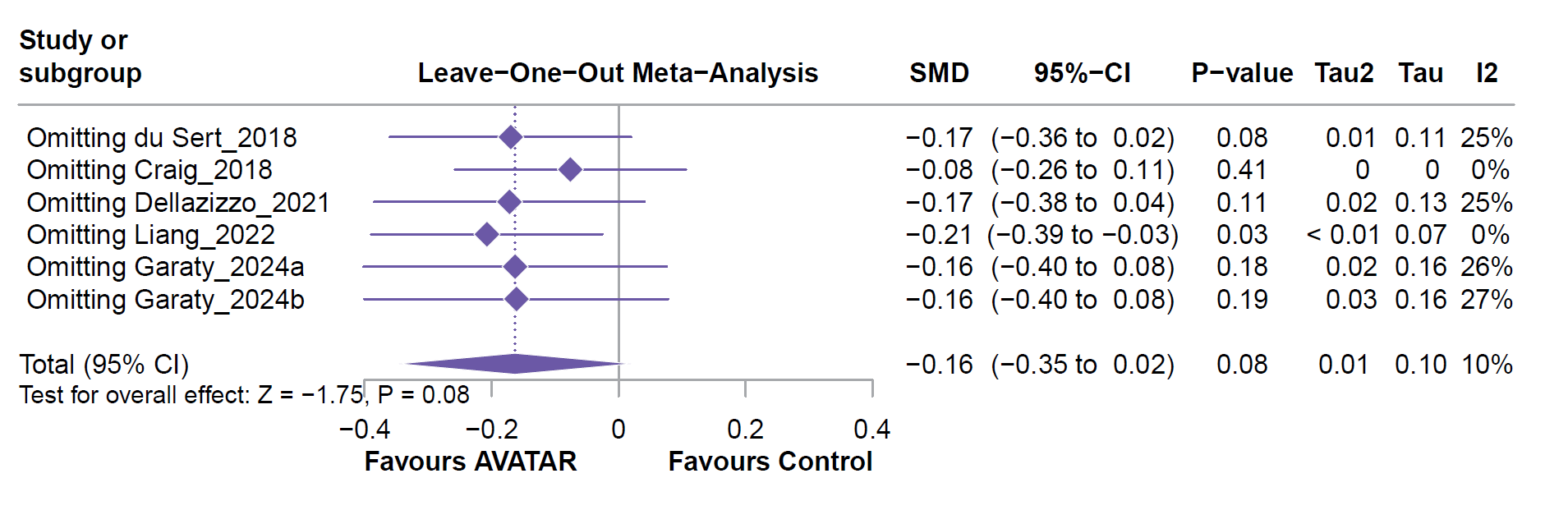


Appendix 1. PRISMA

**Supplement 1**. Checklist of PRISMA guideline

| **Section and Topic** | **Item #** | **Checklist item** | **Location where item is reported** |
| --- | --- | --- | --- |
| **TITLE** | | |  |
| Title | 1 | Identify the report as a systematic review. | title |
| **ABSTRACT** | | |  |
| Abstract | 2 | See the PRISMA 2020 for Abstracts checklist. | Abstract section |
| **INTRODUCTION** | | |  |
| Rationale | 3 | Describe the rationale for the review in the context of existing knowledge. | 1st -2nd paragraph of introduction |
| Objectives | 4 | Provide an explicit statement of the objective(s) or question(s) the review addresses. | 3rd paragraph of introduction |
| **METHODS** | | |  |
| Eligibility criteria | 5 | Specify the inclusion and exclusion criteria for the review and how studies were grouped for the syntheses. | paragraph of " Eligibility Criteria" of methods |
| Information sources | 6 | Specify all databases, registers, websites, organisations, reference lists and other sources searched or consulted to identify studies. Specify the date when each source was last searched or consulted. | paragraph of " Data Sources and Search " of methods  Appendix 2 |
| Search strategy | 7 | Present the full search strategies for all databases, registers and websites, including any filters and limits used. | paragraph of " Data Sources and Search " of methods  Appendix 2 |
| Selection process | 8 | Specify the methods used to decide whether a study met the inclusion criteria of the review, including how many reviewers screened each record and each report retrieved, whether they worked independently, and if applicable, details of automation tools used in the process. | paragraph of “study selection” of methods  Appendix 2 and 3 |
| Data collection process | 9 | Specify the methods used to collect data from reports, including how many reviewers collected data from each report, whether they worked independently, any processes for obtaining or confirming data from study investigators, and if applicable, details of automation tools used in the process. | paragraph of " Data Extraction and Outcome Definition " of methods |
| Data items | 10a | List and define all outcomes for which data were sought. Specify whether all results that were compatible with each outcome domain in each study were sought (e.g. for all measures, time points, analyses), and if not, the methods used to decide which results to collect. | paragraph of " Data Extraction and Outcome Definition " of methods |
|  | 10b | List and define all other variables for which data were sought (e.g. participant and intervention characteristics, funding sources). Describe any assumptions made about any missing or unclear information. | paragraph of " Data Extraction and Outcome Definition " of methods |
| Study risk of bias assessment | 11 | Specify the methods used to assess risk of bias in the included studies, including details of the tool(s) used, how many reviewers assessed each study and whether they worked independently, and if applicable, details of automation tools used in the process. | paragraph of " Quality Assessment" of methods |
| Effect measures | 12 | Specify for each outcome the effect measure(s) (e.g. risk ratio, mean difference) used in the synthesis or presentation of results. | paragraph of " Data synthesis" of methods |
| Synthesis methods | 13a | Describe the processes used to decide which studies were eligible for each synthesis (e.g. tabulating the study intervention characteristics and comparing against the planned groups for each synthesis (item #5)). | paragraph of " Data synthesis" of methods |
|  | 13b | Describe any methods required to prepare the data for presentation or synthesis, such as handling of missing summary statistics, or data conversions. | paragraph of " Data synthesis" of methods |
|  | 13c | Describe any methods used to tabulate or visually display results of individual studies and syntheses. | paragraph of " Data synthesis" of methods |
|  | 13d | Describe any methods used to synthesize results and provide a rationale for the choice(s). If meta-analysis was performed, describe the model(s), method(s) to identify the presence and extent of statistical heterogeneity, and software package(s) used. | paragraph of " Data synthesis" of methods |
|  | 13e | Describe any methods used to explore possible causes of heterogeneity among study results (e.g. subgroup analysis, meta-regression). | paragraph of " Data synthesis" of methods |
|  | 13f | Describe any sensitivity analyses conducted to assess robustness of the synthesized results. | paragraph of " Data synthesis" of methods |
| Reporting bias assessment | 14 | Describe any methods used to assess risk of bias due to missing results in a synthesis (arising from reporting biases). | Not applicable |
| Certainty assessment | 15 | Describe any methods used to assess certainty (or confidence) in the body of evidence for an outcome. | Not applicable |
| **RESULTS** | | |  |
| Study selection | 16a | Describe the results of the search and selection process, from the number of records identified in the search to the number of studies included in the review, ideally using a flow diagram. | eFigure 1 (PRISMA 2020 flow diagram) |
|  | 16b | Cite studies that might appear to meet the inclusion criteria, but which were excluded, and explain why they were excluded. | Appendix 3 |
| Study characteristics | 17 | Cite each included study and present its characteristics. | eTable 1 |
| Risk of bias in studies | 18 | Present assessments of risk of bias for each included study. | paragraph of " Quality of evidence" of result  efigure 2-3 |
| Results of individual studies | 19 | For all outcomes, present, for each study: (a) summary statistics for each group (where appropriate) and (b) an effect estimate and its precision (e.g. confidence/credible interval), ideally using structured tables or plots. | 1^st^ paragraph of result  etable 1 |
| Results of syntheses | 20a | For each synthesis, briefly summarise the characteristics and risk of bias among contributing studies. | 1^st^ – 2^nd^ paragraph of result |
|  | 20b | Present results of all statistical syntheses conducted. If meta-analysis was done, present for each the summary estimate and its precision (e.g. confidence/credible interval) and measures of statistical heterogeneity. If comparing groups, describe the direction of the effect. | 3rd-5th paragraph of result  Figure 2-4 |
|  | 20c | Present results of all investigations of possible causes of heterogeneity among study results. | 3rd-5th paragraph of result  Figure 2-4 |
|  | 20d | Present results of all sensitivity analyses conducted to assess the robustness of the synthesized results. | Not applicable |
| Reporting biases | 21 | Present assessments of risk of bias due to missing results (arising from reporting biases) for each synthesis assessed. | Not applicable |
| Certainty of evidence | 22 | Present assessments of certainty (or confidence) in the body of evidence for each outcome assessed. | Not applicable |
| **DISCUSSION** | | |  |
| Discussion | 23a | Provide a general interpretation of the results in the context of other evidence. | 1^st^ paragraph of discussion |
|  | 23b | Discuss any limitations of the evidence included in the review. | 6^th^ paragraph of discussion |
|  | 23c | Discuss any limitations of the review processes used. | 6^th^ paragraph of discussion |
|  | 23d | Discuss implications of the results for practice, policy, and future research. | Conclusions section |
| **OTHER INFORMATION** | | |  |
| Registration and protocol | 24a | Provide registration information for the review, including register name and registration number, or state that the review was not registered. | PROSPERO (CRD42025639959) |
|  | 24b | Indicate where the review protocol can be accessed, or state that a protocol was not prepared. | PROSPERO (CRD42025639959) |
|  | 24c | Describe and explain any amendments to information provided at registration or in the protocol. | None |
| Support | 25 | Describe sources of financial or non-financial support for the review, and the role of the funders or sponsors in the review. | None |
| Competing interests | 26 | Declare any competing interests of review authors. | None |
| Availability of data, code and other materials | 27 | Report which of the following are publicly available and where they can be found: template data collection forms; data extracted from included studies; data used for all analyses; analytic code; any other materials used in the review. | Not applicable |

Appendix 2. Complete search strategies

Pubmed (n=33)

(voice* OR auditory hallucination) AND (virtual real* OR AVATAR OR virtual environ* OR virtual character*) with “clinical trial” restriction

CENTRAL (n=158)

(voice* OR auditory hallucination) AND (virtual real* OR AVATAR OR virtual environ* OR virtual character*) with “clinical trial” restriction

Medline (n=296)

(voice* OR auditory hallucination) AND (virtual real* OR AVATAR OR virtual environ* OR virtual character*)

Embase (n= 327)

(voice* OR auditory hallucination) AND (virtual real* OR AVATAR OR virtual environ* OR virtual character*)

PsyInfo (n=17)

(voice* OR auditory hallucination) AND (virtual real* OR AVATAR OR virtual environ* OR virtual character*)

Appendix 3. Reasons for exclusion

**Duplicated data (n=5)**

1. ChiCtr. Virtual reality-assisted therapy for auditory verbal hallucinations: a multicenter randomized controlled clinical trial. https://trialsearchwhoint/Trial2aspx?TrialID=ChiCTR1900027254. 2019.

2. Isrctn. AVATAR_VRSocial therapy for auditory verbal hallucinations in early psychosis. https://trialsearchwhoint/Trial2aspx?TrialID=ISRCTN35980117. 2023.

3. Nct. Virtual Reality Therapy for Treatment-resistant Auditory Hallucinations in Schizophrenia. [https://clinicaltrialsgov/show/NCT03148639. 2017](https://clinicaltrialsgov/show/NCT03148639.%202017).

4. Nct. Avatar Therapy in Comparison to Cognitive Behavioral Therapy for Treatment-resistant Schizophrenia. https://clinicaltrialsgov/show/NCT03585127. 2018.

5. Rus-Calafell M, Ehrbar N, Ward T, Edwards C, Huckvale M, Walke J, et al. Participants' experiences of AVATAR therapy for distressing voices: a thematic qualitative evaluation. BMC psychiatry. 2022;22(1):356.

**No outcome of interest (n=3)**

1. Fleming M, Olsen D, Stathes H, Boteler L, Grossberg P, Pfeifer J, et al. Virtual reality skills training for health care professionals in alcohol screening and brief intervention. Journal of the American Board of Family Medicine. 2009;22(4):387‐98.

2. Freeman D, Lambe S, Galal U, Yu LM, Kabir T, Petit A, et al. Agoraphobic avoidance in patients with psychosis: severity and response to automated VR therapy in a secondary analysis of a randomised controlled clinical trial. Schizophrenia research. 2022;250:50‐9.

3. Gaither ML, Bassett ED, Wilson AL, Marder SR, Bradford DW, Bernard JD, et al. Implementation of Cognitive Behavioral Therapy for psychosis via telehealth: An expert consultation and clinical service model. Psychological Services. 2024;21(3):461-72.

**No outcomes reported (n=18)**

1. Actrn. Virtual Reality Integrated Social Recovery therapy for Young People with Early Psychosis. https://trialsearchwhoint/Trial2aspx?TrialID=ACTRN12624000234516. 2024.

https://trialsearchwhoint/Trial2aspx?TrialID=ACTRN12624000435583. 2024.

2. ChiCtr. Psychotherapy of auditory hallucinations in schizophrenia under digital Avarta. https://trialsearchwhoint/Trial2aspx?TrialID=ChiCTR2100053045. 2021.

3. Irct20220226054121N. AVATAR therapy in refractory auditory hallucinations. https://trialsearchwhoint/Trial2aspx?TrialID=IRCT20220226054121N1. 2022.

4. Isrctn. Optimising AVATAR therapy for distressing voices. https://trialsearchwhoint/Trial2aspx?TrialID=ISRCTN55682735. 2020.

5. Kct. Safety and Efficacy of virtual reality therapy for idea of reference in patients with psychosis and verification of changes in brain connectivity with fMRI.

https://trialsearchwhoint/Trial2aspx?TrialID=KCT0007710. 2022.

6. Kct. Safety and efficacy of virtual reality mindfulness in patients with psychosis. https://trialsearchwhoint/Trial2aspx?TrialID=KCT0007718. 2022.

7. Kroczek LOH, Roßkopf S, Stärz F, Blau M, van de Par S, Mühlberger A. The influence of affective voice on sound distance perception. Journal of Experimental Psychology: Human Perception and Performance. 2024;50(9):918-33.

8. Ku J, Cho W, Kim JJ, Peled A, Wiederhold BK, Wiederhold MD, et al. A virtual environment for investigating schizophrenic patients' characteristics: assessment of cognitive and navigation ability. Cyberpsychology & behavior. 2003;6(4):397‐404.

9. Nct. Comparaison of Avatar Therapy to Cognitive Behavioral Therapy in Schizophrenia With Treatment Refractory Hallucinations. https://clinicaltrialsgov/ct2/show/NCT04054778. 2019.

10. Nct. Virtual Reality Avatar Therapy for People Hearing Voices. https://clinicaltrialsgov/show/NCT04099940. 2019.

11. Nct. CHALLENGE. A Randomised Clinical Trial Examining Virtual Reality Therapy. https://clinicaltrialsgov/ct2/show/NCT04661163. 2020.

12. Nct. Virtual Reality Therapy for Voice Hearing (VR-VOICES): a Randomized Controlled Trial. https://clinicaltrialsgov/ct2/show/NCT06013748. 2023.

13. Nct. Avatar-mediated Therapy Versus Cognitive Behavioural Therapy for Persisting Experiences of Hearing Voices. https://clinicaltrialsgov/ct2/show/NCT05982158. 2023.

14. Nct. Influence of Virtual Reality on Voice Perception and Production. https://clinicaltrialsgov/show/NCT05774457. 2023.

15. Nct. Virtual Reality Experiences as Tools to Support Mental Health in Parents of Children With Autism. https://clinicaltrialsgov/show/NCT05852795. 2023.

16. Nct. Enhancing Psychiatric Nursing Skills in Auditory Hallucination Assessment Via AR/VR and Virtual OSCE. https://clinicaltrialsgov/ct2/show/NCT06605781. 2024.

17. Nct. Neuro-VR: augmenting a Virtual Reality-based Therapy with Neurofeedback for Auditory Hallucinations. https://clinicaltrialsgov/ct2/show/NCT06628323. 2024.

18. Nl O. Virtual reality therapy for voice hearing: a randomized controlled trial. https://trialsearchwhoint/Trial2aspx?TrialID=NL-OMON52102. 2022.

**Not RCT based on AVATAR therapy (n=3)**

1. Lee BM, Kim SW, Lee BJ, Won SH, Park YH, Kang CY, et al. Effects and safety of virtual reality-based mindfulness in patients with psychosis: a randomized controlled pilot study. Schizophrenia. 2023;9(1).

2. O'Brien C, Rus-Calafell M, Craig TK, Garety P, Ward T, Lister R, et al. Relating behaviours and therapeutic actions during AVATAR therapy dialogue: an observational study. The british journal of clinical psychology. 2021;60(4):443‐62.

3. Stefaniak I, Sorokosz K, Janicki A, Wciorka J. Therapy based on avatar-therapist synergy for patients with chronic auditory hallucinations: a pilot study. Schizophrenia research. 2019;211:115‐7.

**Conference abstract, protocol or review (n=17)**

1. Beaudoin M, Potvin S, Dumais A. Comparison of VR-Assisted Therapy to Cognitive- Behavioral Therapy in the treatment of verbal hallucinations in patient with treatment-resistant schizophrenia. European psychiatry. 2023;66:S127.

2. Craig T, Garety P, Ward T, Edwards C, Rus-Calafell M, Huckvale M, et al. The UK AVATAR 1 and 2 Trials for People with Distressing Voices - Findings and Learning from AVATAR1, and AVATAR2 Developments in Theory and Therapy. European psychiatry. 2022;65:S22‐S3.

3. Craig T, Garety P, Ward T, Rus-Calafell M, Williams G, Huckvale M, et al. Computer assisted therapy for auditory hallucinations: the avatar clinical trial. Schizophrenia research. 2014;153:S74.

4. Craig TK, Rus-Calafell M, Ward T, Fornells-Ambrojo M, McCrone P, Emsley R, et al. The effects of an Audio Visual Assisted Therapy Aid for Refractory auditory hallucinations (AVATAR therapy): study protocol for a randomised controlled trial. Trials. 2015;16:349.

5. Dellazizzo L, Potvin S, O'Connor K, Dumais A. A randomized controlled trial comparing virtual reality therapy to cognitive behavioral therapy in schizophrenia with treatment refractory hallucinations: preliminary results. Schizophrenia bulletin. 2018;44:S346‐S7.

6. Dzafic I, Spark J, Bell I, Wood S, Lavoie S, Whitford T, et al. PAPER: the hybrid study: integrating neurofeedback, virtual reality, and cognitive behaviour therapy for the treatment of hearing voices. Early intervention in psychiatry. 2023;17:141.

7. Garety P, Edwards CJ, Ward T, Emsley R, Huckvale M, McCrone P, et al. Optimising AVATAR therapy for people who hear distressing voices: study protocol for the AVATAR2 multi-centre randomised controlled trial. Trials. 2021;22(1):366.

8. Garety P, Edwards CJ, Ward T, Emsley R, Huckvale M, McCrone P, et al. Correction: optimising AVATAR therapy for people who hear distressing voices: study protocol for the AVATAR2 multi-centre randomised controlled trial. Trials. 2024;25(1):803.

9. Garety P, Edwards CJ, Ward T, Emsley R, Huckvale M, McCrone P, et al. Correction: optimising AVATAR therapy for people who hear distressing voices: study protocol for the AVATAR2 multi-centre randomised controlled trial. Trials. 2024;25(1):816.

10. Glenthoj L, Smith L, Mariegaard L, Due AS, Christensen A, Christensen M, et al. CHALLENGE and Face Your Fears: virtual Reality Treatment for Auditory Hallucinations and Paranoid Ideations. European psychiatry. 2022;65:S22.

11. Glenthoj LB. PAPER: using immersive virtual reality in treating paranoia and auditory hallucinations. Early intervention in psychiatry. 2023;17:141‐2.

12. Lee BM, Kim SW, Lee BJ, Won SH, Park YH, Kang CY, et al. Effects and safety of virtual reality-based mindfulness in patients with psychosis: a randomized controlled pilot study. Schizophrenia (heidelberg, germany). 2023;9(1):57.

13. Leff J, Williams G, Huckvale M, Arbuthnot M, Leff AP. Avatar therapy for persecutory auditory hallucinations: what is it and how does it work? Psychosis. 2014;6(2):166‐76.

14. Veling W. Fears, Fun and Voices - an update on VR Treatments for Psychosis. European psychiatry. 2022;65:S22.

15. Ward T, Jamieson-Craig T, Garety PA, Rus-Calafell M, McCrone P, Emsley R, et al. A randomized clinical trial of a novel audio-visual assisted therapy aid for refractory auditory hallucinations (AVATAR therapy). Early intervention in psychiatry. 2014;8:42.

16. Skoneczny T, Tyburski E, Kucharska-Mazur J, Samochowiec J. Use of virtual reality in treatment of refractory auditory hallucinations in schizophrenia: a literature review. European psychiatry. 2019;56:S515.

17. Smith LC, Mariegaard L, Vernal DL, Christensen AG, Albert N, Thomas N, et al. The CHALLENGE trial: the effects of a virtual reality-assisted exposure therapy for persistent auditory hallucinations versus supportive counselling in people with psychosis: study protocol for a randomised clinical trial. Trials. 2022;23(1):773.
